# Systemic Measures and Legislative and Organizational Frameworks Aimed at Preventing or Mitigating Drug Shortages in 28 European and Western Asian Countries

**DOI:** 10.3389/fphar.2017.00942

**Published:** 2018-01-18

**Authors:** Tomasz Bochenek, Vafa Abilova, Ali Alkan, Bogdan Asanin, Iñigo de Miguel Beriain, Zeljka Besovic, Patricia Vella Bonanno, Anna Bucsics, Michal Davidescu, Elfi De Weerdt, Natasa Duborija-Kovacevic, Jurij Fürst, Mina Gaga, Elma Gailīte, Jolanta Gulbinovič, Emre U. Gürpınar, Balázs Hankó, Vincent Hargaden, Tor A. Hotvedt, Iris Hoxha, Isabelle Huys, Andras Inotai, Arianit Jakupi, Helena Jenzer, Roberta Joppi, Ott Laius, Marie-Camille Lenormand, Despina Makridaki, Admir Malaj, Kertu Margus, Vanda Marković-Peković, Nenad Miljković, João L. de Miranda, Stanislav Primožič, Dragana Rajinac, David G. Schwartz, Robin Šebesta, Steven Simoens, Juraj Slaby, Ljiljana Sović-Brkičić, Tomas Tesar, Leonidas Tzimis, Ewa Warmińska, Brian Godman

**Affiliations:** ^1^Department of Drug Management, Faculty of Health Sciences, Jagiellonian University Medical College, Krakow, Poland; ^2^Analytical Expertise Centre, Ministry of Health, Baku, Azerbaijan; ^3^Turkish Medicines and Medical Devices Agency, Ankara, Turkey; ^4^Department of Surgery, Department of Medical Ethics, Medical Faculty of the University of Montenegro, Podgorica, Montenegro; ^5^RG Chair in Law and the Human Genome, University of the Basque Country, Leioa, Spain; ^6^Montenegrin Agency for Drugs and Medical Devices, Sector for Drugs and Medical Devices, Podgorica, Montenegro; ^7^Department of Pharmacoepidemiology, Strathclyde Institute of Pharmacy and Biomedical Sciences, University of Strathclyde, Glasgow, United Kingdom; ^8^Mechanism of Coordinated Access to Orphan Medicinal Products, Brussels, Belgium; ^9^Clalit Health Services Headquarters, Tel-Aviv, Israel; ^10^Department of Pharmaceutical and Pharmacological Sciences, KU Leuven, Leuven, Belgium; ^11^Department of Pharmacology and Clinical Pharmacology, Medical Faculty of the University of Montenegro, Podgorica, Montenegro; ^12^Department of Medicines, Health Insurance Institute, Ljubljana, Slovenia; ^13^7th Respiratory Medicine Department, Athens Chest Hospital Sotiria, Athens, Greece; ^14^State Agency of Medicines, Riga, Latvia; ^15^Department of Pathology, Forensic Medicine and Pharmacology, Vilnius University, Vilnius, Lithuania; ^16^State Medicine Control Agency, Vilnius, Lithuania; ^17^University Pharmacy Department of Pharmacy Administration, Semmelweis University, Budapest, Hungary; ^18^School of Mechanical and Materials Engineering, University College Dublin, Dublin, Ireland; ^19^Norwegian Medicines Agency, Oslo, Norway; ^20^Department of Pharmacy, University of Medicine, Tirana, Albania; ^21^Syreon Research Institute, Budapest, Hungary; ^22^Department of Health Policy and Health Economics, Institute of Economics, Eötvös Loránd University, Budapest, Hungary; ^23^Department of Drug Management, Faculty of Pharmacy, UBT (Kosovo), Prishtina, Albania; ^24^Health Department, Bern University of Applied Sciences, Bern, Switzerland; ^25^University Hospital of Psychiatry Zurich (PUK), Zurich, Switzerland; ^26^Local Health Unit of Verona—Veneto Region, Verona, Italy; ^27^State Agency of Medicines, Tartu, Estonia; ^28^CNAMTS, Statutory Health Insurance for Salaried Workers, Paris, France; ^29^Panhellenic Association of Hospital Pharmacists, Athens, Greece; ^30^National Organization for Medicines, Athens, Greece; ^31^Estonian State Agency of Medicines, Tartu, Estonia; ^32^Ministry of Health and Social Welfare, Banja Luka, Republic of Srpska, Bosnia and Herzegovina; ^33^Faculty of Medicine, Department of Social Pharmacy, University of Banja Luka (Republic of Srpska), Banja Luka, Bosnia and Herzegovina; ^34^Institute of Orthopaedic Surgery Banjica, Belgrade, Serbia; ^35^Escola Superior de Tecnologia e Gestão, Instituto Politécnico de Portalegre, Portalegre, Portugal; ^36^Centro de Recursos Naturais e Ambiente, Instituto Superior Técnico, Universidade de Lisboa, Lisboa, Portugal; ^37^Agency for Medicinal Products and Medicinal Devices, Ljubljana, Slovenia; ^38^Clinical Centre of Serbia, Belgrade, Serbia; ^39^Graduate School of Business Administration, Bar-Ilan University, Ramat-Gan, Israel; ^40^State Institute for Drug Control, Prague, Czechia; ^41^Croatian Health Insurance Fund, Zagreb, Croatia; ^42^Department of Organisation and Management in Pharmacy, Pharmaceutical Faculty, Comenius University, Bratislava, Slovakia; ^43^Chania General Hospital, Crete, Greece; ^44^Dentons Europe Dąbrowski i Wspólnicy sp. k., Warszawa, Poland; ^45^Division of Clinical Pharmacology, Karolinska University Hospital, Karolinska Institutet, Stockholm, Sweden; ^46^Health Economics Centre, Liverpool University Management School, Liverpool, United Kingdom

**Keywords:** drug shortage, pharmaceutical policy, health care system, legislation, organizational framework, Europe, European Union, Western Asia

## Abstract

Drug shortages have been identified as a public health problem in an increasing number of countries. This can negatively impact on the quality and efficiency of patient care, as well as contribute to increases in the cost of treatment and the workload of health care providers. Shortages also raise ethical and political issues. The scientific evidence on drug shortages is still scarce, but many lessons can be drawn from cross-country analyses. The objective of this study was to characterize, compare, and evaluate the current systemic measures and legislative and organizational frameworks aimed at preventing or mitigating drug shortages within health care systems across a range of European and Western Asian countries. The study design was retrospective, cross-sectional, descriptive, and observational. Information was gathered through a survey distributed among senior personnel from ministries of health, state medicines agencies, local health authorities, other health or pharmaceutical pricing and reimbursement authorities, health insurance companies and academic institutions, with knowledge of the pharmaceutical markets in the 28 countries studied. Our study found that formal definitions of drug shortages currently exist in only a few countries. The characteristics of drug shortages, including their assortment, duration, frequency, and dynamics, were found to be variable and sometimes difficult to assess. Numerous information hubs were identified. Providing public access to information on drug shortages to the maximum possible extent is a prerequisite for performing more advanced studies on the problem and identifying solutions. Imposing public service obligations, providing the formal possibility to prescribe unlicensed medicines, and temporary bans on parallel exports are widespread measures. A positive finding of our study was the identification of numerous bottom-up initiatives and organizational frameworks aimed at preventing or mitigating drug shortages. The experiences and lessons drawn from these initiatives should be carefully evaluated, monitored, and presented to a wider international audience for careful appraisal. To be able to find solutions to the problem of drug shortages, there is an urgent need to develop a set of agreed definitions for drug shortages, as well as methodologies for their evaluation and monitoring. This is being progressed.

## Introduction

We are accustomed to thinking that commodities produced anywhere across the globe will be available to consumers within a relatively short period of time, if not immediately. Typically, access to sufficient financial resources has been the only obstacle in acquiring these products. Despite this, and surprisingly, in the second decade of the twenty-first century, shortages of pharmaceuticals have increasingly become an issue in many countries for a number of reasons. Drug shortages has been the focus of academic and practitioner research, initially in the USA and Canada, but subsequently in a number of European countries and other continents (Morrison, 2011; Ventola, 2011; Birgli, 2013; McBride et al., 2013; Costelloe et al., 2014; Goldsack et al., 2014; Bogaert et al., 2015; Butterfield et al., 2015; De Weerdt et al., 2015b, 2017b; Pauwels et al., 2015; Alsheikh et al., 2016; Awad et al., 2016; Yang et al., 2016; Heiskanen et al., 2017; Mazer-Amirshahi et al., 2017; Walker et al., 2017). Some medicines are simply not available on the market in certain countries, even if there is sufficient money to pay for them. The scientific evidence underpinning drug shortages, including the extent and rationale, is still scarce. However, the amount of evidence is gradually increasing and the problem is now a permanent feature within the public and scientific discourse. This problem needs to be addressed urgently, especially for critical medicines, in order to avoid any negative impact on patients.

One of the seminal cross-country reports on drug shortages in Europe, including an in-depth analysis of the situation in France, Greece, Poland, Spain, and the United Kingdom, proposed a classification of reasons for shortages into unpredictable and predictable issues (Birgli, 2013). The first category of reasons embraces natural disasters, manufacturing problems, raw material shortages, non-compliance with regulatory standards, packaging shortages, unexpected demand, epidemics, parallel distribution, competitive issues, foreign exchange effect, and sovereignty issues (e.g., a financial crisis). The predictable reasons include: product discontinuation, industry consolidation, limited manufacturing capacity, just-in-time inventories, rationing and quotas, deliberate shortages to manipulate price, market shifts, the launch of a new competitor or formulation, and patent expiry (Birgli, 2013).

The characteristics of drug shortages, such as the assortment or range of non-available products, are different in each country but there are some common themes. In the USA, the majority of shortages were reported to occur among sterile injectable medications (McLaughlin and Skoglund, 2015). In addition, the number of generic medicines experiencing shortages in the USA has risen appreciably in recent years from 154 in 2007 to 456 in 2012 (United States Government Accountability Office Report, 2014). In several European countries, for example Belgium, the Netherlands, the United Kingdom (England), Italy, Germany, Spain, and France, injectable forms dominated reports on shortages in two categories: oncology medicines (79%) and medicines defined as essential by the WHO (52%; Pauwels et al., 2014; World Health Organization, 2017). Drug shortages have an indisputable impact on public health, especially when they cause a delay in starting treatment or difficulty in its continuation, lowering or omitting doses, increasing costs of treatment, selection of patients, or putting an increased administrative burden on health care staff (Ventola, 2011; McLaughlin et al., 2013; Bocquet et al., 2017; De Weerdt et al., 2017b).

According to a survey performed among anesthesiologists in the USA, 90% experienced a problem with shortages of anesthetics (with at least one drug) at the time of the survey, while this increased to 98% in the last year (American Society of Anesthesiologists, 2017). Moreover, 92% linked the shortage with the necessity to use alternative drugs, 6% had to postpone and 4% had to cancel procedures (American Society of Anesthesiologists, 2017). From the hospital pharmacists' perspective, according to a survey on shortages of injectable medicines, the impact of shortages in the USA was significant (Goldsack et al., 2014). As many as 99% of pharmacists reported experiencing at least one drug shortage during the previous 12 months, 64% reported that their facility had completely run out of at least one injectable oncology drug during that period of time and 25% reported that one or more safety events had occurred at their facility as a result of drug shortages (Goldsack et al., 2014). Shortages were forcing hospitals to apply various management strategies–83% of respondents reported that providers may have changed the treatment of their patients as a result of drug shortages, 43% reported treatment delays and 38%—the prioritization of patients for treatment based on clinical factors. Moreover, shortages of injectable oncology drugs had a direct impact on treatment costs: for example, 74% of respondents reported that their facility had received an offer to purchase drugs in short supply at a higher price, and 65% reported that overall treatment costs had increased due to drug shortages. The major cost drivers were primarily: increased labor spending, expansion of inventory levels, purchasing of more expensive (branded or generic) substitute drugs, and purchasing of a drug in short supply from an alternate supplier at a higher price (Goldsack et al., 2014).

Similar to the USA, drug shortages have also been reported in European countries to have a serious impact on health care systems and public health [European Association of Hospital Pharmacists (EAHP) secretariat, 2014; Pauwels et al., 2015]. A large pan-European survey on medicines supply shortages in the hospital sector, their prevalence, nature, and impact on patient care, revealed that 75% of responders (hospital pharmacists) agree or strongly agree that shortages had a negative impact on patient care in their hospitals [European Association of Hospital Pharmacists (EAHP) secretariat, 2014]. The majority of hospital pharmacists, responding to questions in another pan-European survey, indicated increased hospital costs, pharmacy or personnel costs, and the use of more expensive alternatives as frequently or always occurring consequences of drug shortages (Pauwels et al., 2015). As far as the level of personnel stress was concerned, 37% of respondents indicated that drug shortages influenced it very severely. The total time spent on the management of drug shortages was estimated to be 13 h per week (Pauwels et al., 2015). Belgian hospital pharmacists spent a median of 109 min a week on drug supply problems, carrying out 59% of the total time spent on these problems in their hospitals and being supported by pharmacy technicians (27% of the total time), and logistic or administrative personnel (De Weerdt et al., 2017a). The Flemish community pharmacists spent approximately half an hour per week on drug supply problems, mainly checking missing products from orders, contacting wholesalers or manufacturers regarding potential drug shortages and communicating to patients (De Weerdt et al., 2017c).

Drug shortages can also be considered as ethical and political issues. They threaten the capacity for clinicians and governments to fulfill their moral obligations to patients and society associated with providing benefit, minimizing harm and promoting equity, especially in Europe. Moreover, they stem from societal values, especially from the choices that societies have made about what they want most from pharmaceutical industries, regulators, and health services (Lipworth and Kerridge, 2013). In the USA, the challenge was explicitly expressed as “No more denying. You are in denial too if you believe that this country's pharmaceutical industry (…) can reliably supply medications for patients.” (Wenzel, 2015). There is an ethical imperative to prevent drug shortages. However, these shortages stem partly from choices that societies make about how they want to organize their markets, health care services and regulatory environment and, for that reason, any proposed solution will likely threaten the various stakeholder groups' values and require moral trade-offs, difficult choices within health care systems, and reordering priorities (Lipworth and Kerridge, 2013; Schweitzer, 2013).

The focus of our previous study was analyzing, characterizing and assessing drug shortages in Belgium and France, while also adopting a wider perspective from the European Union (EU). We identified and addressed four major themes: (a) defining drug shortages, (b) their dynamics and perception, (c) their determinants, and (d) the role of the European and national institutions in coping with the problem (Bogaert et al., 2015). We found that there are three major groups of determinants to this problem: manufacturing problems, distribution and supply problems, and problems related to economic aspects. The EU Member States are striving to resolve this problem very much on their own, although there is an initiative run by the European Medicines Agency (EMA) whereby a Shortages Catalogue is being maintained by the EMA (European Medicines Agency, 2017). A far more focused and dedicated collaboration may well prove instrumental in coping more effectively with drug shortages.

Learning from other countries' experiences should not be underestimated or underutilized in shaping local or national pharmaceutical policies (Godman et al., 2010, 2014; Vončina et al., 2011; Malmström et al., 2013; Moon et al., 2014; Ferrario et al., 2017). There could be lessons to be drawn from cross-country comparisons, even if a given country's characteristics do not perfectly correspond in terms of geographical location, size, demography, economy, or type of health care system. Consequently, the objective of the current study was to characterize, compare, and evaluate the current systemic measures, legislation, and legislative frameworks aimed at preventing or mitigating drug shortages existing within health care systems across a wide range of European and Western Asian countries.

## Materials and methods

The design of this study was retrospective, cross-sectional, descriptive, and observational. To achieve the objective of this study, a survey form was prepared. It contained questions pertaining to: (1) general characteristics of drug shortages; (2) alertness to drug shortages and a description of the information systems to capture shortages; (3) public service obligations; and (4) regulations associated with the problem of drug shortages. Full information on content of a survey form, including all detailed questions, can be found as Supplementary Material. The survey form was pilot-tested on five international pharmaceutical market professionals before being issued. Information was gathered through an interactive, iterative process. Written responses to the survey questions were given by the co-authors, who are typically senior health authority, health insurance company personnel or their advisers, and were knowledgeable about the current situation in the national pharmaceutical markets of all the included countries. They represented personnel from ministries of health, state medicines agencies, local health authorities, other health or pharmaceutical pricing and reimbursement authorities, health insurance companies and academic institutions. The responses to survey questions were verified for accuracy and appropriate understanding of the country-specific arrangements by checking documents, legal acts, and regulations pertinent to the studied problems. They were re-checked and re-confirmed with the co-authors to enhance the robustness of the findings and potential ways forward.

To further enhance the robustness of the study results, the published sources, including the scientific literature, legal acts and information gathered and disclosed within the public domain by organizations involved in pharmaceutical markets of the studied countries, were used as well. The methodological strategy used in this study was case-oriented, seeking to better understand the dynamics of the global problem of drug shortages, based on a number of cases selected from countries of Europe and Western Asia, characterized by different epidemiologies, geographies, GDPs per capita, levels of spending on health care, and approaches to the pricing of medicines (Cacace et al., 2013). The potential respondents representing all 28 member countries of the EU and 4 of the European Free Trade Association (EFTA), as well as 10 non-EU/EFTA countries were invited to participate in this study. Respondents from 14 countries either declined to participate or returned incomplete surveys which could not be improved or corrected. The response rate was 67%. Therefore, the overall number of the included countries was 28 and this group consisted of 20 EU/EFTA countries and 8 non-EU/EFTA countries. This cross-country comparative study reflects the situation as in spring 2017.

The policy document analysis approach was applied in this study, and no interviews, requiring recruitment and obtaining informed consent from humans were conducted. Information that can be disclosed to the public and/or is accessible in the public domain was sought in this study. Consequently, ethics approval was not required and the study has no ethical implications associated with its design and conduct.

## Results

### Definitions, occurrence, and dynamics of drug shortages

Formal and legally binding definitions of drug shortages currently do not exist in the majority of the studied countries, with the exception of Belgium, France, Italy, and Spain, where the most comprehensive descriptions were coined. The situation of the unavailability of medicines on the Belgian market is defined as follows: “A drug is unavailable when enterprises that are responsible for the marketing of the drug are unable to deliver that drug for an uninterrupted period of four consecutive days to the community pharmacies, hospital pharmacies or wholesalers in Belgium^1^.” Moreover, a second definition was formulated through regulations for reporting unavailability: “Holders of the market authorization should notify the Federal Agency of Medicines and Health Products (FAMHP) when a drug will be unavailable for a time period longer than 14 days. The notification should be made within 7 days after the start of the unavailability” (De Weerdt et al., 2015a). A drug shortage is defined by law in France as an inability for a community pharmacy or a hospital pharmacy to deliver a drug within 72 h^2^ Additionally, drug shortages in France have been classified formally into two separate contexts of either stock or supply problems. A stock-related shortage is defined as the lack of possibility to manufacture a medicine, whereas a supply-related shortage is defined as a problem in the distribution chain that makes the supply of a medicine impossible, even if enough of the medicine has been manufactured^2^. A formal, legal description of drug shortages also exists in Italy. The Italian Medicines Agency (AIFA) defines medicines in short supply as: “Medicines which are not available or not to be found on the whole Italian market, because the marketing authorization holder (MAH) is unable to guarantee the correct and regular supply to meet patients' needs^3^”.

Although in Spain there was no single standard definition established for the whole country (which was reported as a rather serious problem in monitoring shortages), several definitions were coined by different entities. The Spanish Agency for Medicines and Health Products (Spanish acronym: AEMPS), being part of the Spanish Ministry of Health Care, defined the “supply problem” as a situation in which the number of available units of a drug in the pharmaceutical trade channel is below the level of national or local consumption needs, being often due to problems in the manufacturing or distribution of a drug^4^. The Regional Government of Madrid defined the “supply problem” as a continued and widespread shortage of a drug in pharmacies that may be due to problems in manufacturing, procurement of raw materials or distribution^5^. The Government of Valencia approved a regulation in 2008 where “insufficient supply” was delineated very precisely. It allows the Department of Health to proclaim the state of “insufficient supply,” in order to avoid serious problems with supply of medicines or their shortages. Proclamation is based on signals gathered from the pharmaceutical market, observations of processing of drug supply orders and frequency of substitution of prescriptions for a particular drug. All this information is reported through a pharmaceutical information system named Gaia^6^.

Two descriptions of situations associated with drug shortages currently exist in Greece (actual shortages and temporary interruptions in supply), although a coherent, official definition, considering the duration of a shortage, does not currently exist in the country. The situation of an actual drug shortage pertains to the lack of capability to fulfill the demand and the non-availability of a drug in the whole health care system, without the possibility to obtain that medicine from any source. Interruptions in supply refer to situations when drugs are not commercially available, mainly for commercial reasons, for a limited time duration.

In the rest of the studied countries, only indirect formal descriptions of situations pertaining to the problem of drug shortages were found, but they cannot be considered as straightforward definitions. They are associated with the revocation of marketing authorization (MA) in cases of not placing a product on the market, not responding to requests of supply from hospitals, as well as situations of suspending distribution or disrupting supplies of medicines.

Interestingly, in Hungary, “drug shortage” as a term is reported to be widely used in the legislation, including the act requiring the MAHs to report in case they are not able to supply^7^, but without any association with a concrete formal definition. A similar situation was found in Norway, where there is also no formal definition; a temporary disruption of a medicine's marketing was de facto considered to be a shortage as soon as it lasted for at least 2 weeks. In the Croatian legislation, the closest term in meaning related to drug shortages is “disturbance on the medicines' market.” Drug shortages are not formally defined in Israel, but various health care institutions, including Health Maintenance Organizations (HMO) or private pharmacies, define shortages according to their own needs. For example, in the Clalit Health Services (the largest state-mandated HMO), shortages are considered as a stock covering <1 month's expected consumption, whilst for other institutions it might be different, depending on the profile of customers, their needs, and logistical considerations. Similarly, in Switzerland, the four major public and private organizations gathering information on drug shortages and bottlenecks in the supply of drugs use different descriptions of drug shortages, depending on the mission and strategic goals of these organizations. Consequently, these definitions are associated with focusing on restricted, as compared to usual, availability (as in case of the Federal Office of Public Health—FOPH); the essential role in pharmacological treatment (Swiss Agency for Therapeutic Products—Swissmedic, i.e., the state drug registration agency, as well as the Federal Office for National Economic Supply—FONES); the duration of the disruption exceeding 14 days and lack of availability of all doses and package sizes (FONES); or supplies not satisfying demand and orders (Martinelli Consulting)^8^^,^ ^9^^,^ ^10^^,^ ^11^.

According to the respondents, drug shortages have been occurring in all of the studied countries throughout the last decade, and typically have been increasing. This is similar to the situation in the USA (United States Government Accountability Office Report, 2014). Drug shortages were often reported as “always present,” with a starting point difficult to set in time. For some countries, the following breakthrough timeframes or starting points of the problem have been elicited from the respondents as: the early nineties of the twentieth century (Estonia, Montenegro, Serbia, Slovenia), 2006 (France), around 2007 (Greece, Switzerland), between 2009 and 2011 (Austria, Slovakia), between 2011 and 2012 (Spain), 2012 (Hungary, Poland), 2013 (Italy), and 2015 (Azerbaijan, Israel).

In Albania, the drug shortages in the past (especially until the early 1990s) were a much more serious problem than nowadays; being a consequence of structural issues characterizing the centralized pharmaceutical market. In Azerbaijan, where there is no compulsory health care insurance (planned for the near future), but the state provides state hospitals and 25 (out of 2,240) preferential pharmacies executing state programs with necessary medicines, the list of publicly funded medicines is approved by the Ministry of Health (MoH) and the problem of shortages does not pertain to this list. However, shortages have been noted in the case of medicines distributed in the private sector in Azerbaijan. The description of the situation in Kosovo is complicated since, due to the war of 1999 and donations from different sources, drugs were being initially registered through the provisional MA procedure, replaced in 2006 by the regular MA at the Kosovo Medicines Agency. Nevertheless, there are still a certain number of medicines without MA (due to the small size of the country, very limited budget and spending for health care) and, as such, drug shortages are still evident in Kosovo. As a result of further changes in the legislation, drug shortages started decreasing from 2013 and the formerly very poor situation is now improved.

The dynamics of medicines shortages in the past 3 years have been reported very differently for the studied countries. These were increasing in France, Greece, Italy, Latvia, Lithuania, Norway, Ireland, Israel, Slovakia, Switzerland, and Turkey, slightly increasing in the Czech Republic, while remaining stable in Croatia, Serbia, and Estonia. In the latter two countries, as well as in Hungary, the problem started to be more intensely reported during the past 3 years for administrative reasons, so it is not clear to what extent the shortages were really increasing and to what extent their reporting more accurately reflected an existing situation. In Belgium, the increased awareness of MAH on reporting drug shortages was in parallel with the increased reporting of hospital pharmacists and direction of shortages' trend, which seemed to be increasing. The drug shortages' dynamics were formerly increasing, but have recently decreased, in Poland and Spain, and have been decreasing in Slovenia. In Austria, Albania, Azerbaijan and the Republic of Srpska (Bosnia and Herzegovina; BIH) and Montenegro, the dynamics were characterized as difficult or impossible to assess, while remaining unclear but reported cautiously (because of the potential reporting bias mentioned above) as increasing in Hungary.

### Information systems and vigilance related to drug shortages

The existence of publicly available databases on drug shortages has been reported from the majority of studied countries, while in some countries access to the gathered information was not fully open or was limited to the public (Albania, Azerbaijan, the Czech Republic, Montenegro, and Serbia). Apparently, publicly available databases exist in almost all of the studied EU/EFTA countries, except for the Czech Republic. Therefore, also the quality of any publicly revealed statistics on drug shortages in the Czech Republic is described as difficult to assess and drug shortages are not analyzed systematically. In Turkey, information on shortages of medicines used only in hospital settings, is made available only to the selected relevant stakeholders and only part of the registry is made publicly available. In Albania, information on shortages can be made available to a requesting party—but only on special demand. Full information on the publicly available databases on drug shortages (including the frequency of their updating and other characteristics that were identified in the studied countries) can be found in Tables 1, 2. The highest number of publicly available databases (four) was revealed in Switzerland. The content of these databases was found to be differentiated depending on the purposes set by the organizations running these databases.

**Table 1:** Publicly available databases on drug shortages in countries of the European Union and the European Free Trade Association.

**Country**	**Organization in charge of database**	**Access**	**Frequency of database updating**	**Notes**
Austria	Austrian Medicines and Medical Devices Agency (AGES MEA)	www.basg.gv.at/news-center/news/news-detail/article/uebersichtsliste-vertriebseinschraenkungen-986/	Weekly	–
Belgium	Federal Agency of Medicines and Health Products	https://www.fagg-afmps.be/nl/items-HOME/Onbeschikbaarheid_van_geneesmiddelen	Daily (this is nominal frequency, which actually may be different)	–
Croatia	Croatian Health Insurance Fund	http://www.hzzo.hr/zdravstveni-sustav-rh/trazilica-za-lijekove-s-vazecih-lista/	Monthly	Database known as “Disturbance on Market of Drugs” (“Poremećaj opskrbe tržišta lijekovima”). Information on shortages is limited to reimbursed medicines only.
Estonia	Estonian State Agency of Medicines	http://www.ravimiamet.ee/ulevaatlik-tabel-humaanravimite-tarneraskustest	As necessary (after every notification by MAH)	Information on shortages covers all medicines marketed in Estonia.
France (1)	French Agency for Medicines Safety (ANSM—Agence Nationale pour la Sécurité du Medicament)	http://ansm.sante.fr/Mediatheque/Publications/Information-in-English	Yearly	–
France (2)	National Council of the College of Pharmacists (Conseil national de l'ordre national des pharmaciens—CNOP)	http://www.ordre.pharmacien.fr/Le-Dossier-Pharmaceutique/Ruptures-d-approvisionnement-et-DP-Ruptures	Monthly	–
Greece	National Organization for Medicines (EOF)	http://www.eof.gr/web/guest/eparkeia	Monthly (this is usual frequency)	Database contains the following information: medicine's code, brand name, pharmaceutical form, active substance, therapeutic category, estimated duration of the shortage, alternative substances, information regarding if the medicine is imported by the Institution for Pharmaceutical Research and Technology (IFET, a public company).
Hungary	National Institute of Pharmacy and Nutrition	https://www.ogyei.gov.hu/temporary_discontinuation_of_sale_/	Weekly	Database known as “Temporary Drug Shortages Database.” Contains records from 2010.
Ireland (1)	Irish Pharmaceutical Union (IPU)	https://ipu.ie/home/ipu-product-file/medicine-shortages/	Minimum monthly, but on demand based on completion of medicines shortages notification form	Database available for download as pdf. Details include company, product, pack size, GMS No, availability. IPU Medicines Shortages notification form available for download. On completion, e-mail address to send the form is provided. Database is then updated.
Ireland (2)	UniPhar	www.uniphar.ie	As needed	Access is limited to pharmacists after sign-in (online platform run by wholesaler).
Italy	Italian Medicines Agency (AIFA—Agenzia Italiana del Farmaco)	http://www.aifa.gov.it/content/carenze-e-indisponibilt%C3%A0	Weekly	–
Latvia	State Agency of Medicines (SAMLV)	https://www.zva.gov.lv//?id=781&lang=&top=334&sa=673	Daily	–
Lithuania	State Medicines Control Agency (SMCA)	www.vvkt.lt	Biweekly	Database contains raw information and announcements on drug shortages; without processed statistics.
Malta (1)	Ministry for Health (CPSU—Central Procurement and Supplies Unit)	https://health.gov.mt/en/cpsu/Pages/POYC-OOS.aspx	Weekly	Database known as “Pharmacy of Your Choice (POYC) Out of Stock Information.” Information on out-of-stock medicines (covers only medicines supplied through the public health system).
Malta (2)	Ministry for Health (CPSU—Central Procurement and Supplies Unit)	https://health.gov.mt/en/cpsu/Pages/Items-Problematic-To-Source.aspx	As required	Database known as “Items Problematic to Source.” List of items that CPSU has found problematic to source (covers only medicines supplied through the public health system).
Norway	Norwegian Medicines Agency	https://legemiddelverket.no/legemiddelmangel/legemiddelmangel-og-avregistreringer-2017-rad-til-apotek-og-helsepersonell	Weekly	–
Poland	Ministry of Health	http://www.bip.mz.gov.pl/legislacja/akty-prawne/obwieszczenie-ministra-zdrowia-z-dnia-10-lipca-2017-r-w-sprawie-wykazu-produktow-leczniczych-srodkow-spozywczych-specjalnego-przeznaczenia-zywieniowego-oraz-wyrobow-medycznych-zagrozonych-brakiem-do/	At least bimonthly	Database known as “List of Medicinal Products, Food Products for Special Dietary Use and Medical Devices Vulnerable to Lack of Availability on Territory of the Republic of Poland.” Any of the listed products can be banned from export from Poland, considering the threat to its availability on the national market and significance to public health (main reason for establishing the list).
Portugal (1)	Portugal ANF—National Association of Pharmacies (ANF—Associação Nacional de Farmécias)	https://www.anfonline.pt/	Daily	The ANF members (legal owners of community pharmacies) update the database with daily regularity, through the information system managed by Center for Health Research and Evaluation CEFAR (Centro de Estudos e Avaliação em Saúde). These daily updates are then aggregated by ANF and INFARMED into weekly and monthly reports and newsletters.
Portugal (2)	INFARMED—National Authority of Medicines and Health Products (Autoridade Nacional do Medicamento e Produtos de Saúde, I.P.)	www.infarmed.pt	Daily	As above.
Slovakia	The State Institute for Drug Control (SUKL)	http://www.sukl.sk/en/inspection/post-authorization-quality-control/export-of-medicinal-products/list-of-medicinal-products-for-which-they-were-issued-decisions-not-to-allow-the-export-from-slovak-republic?page_id=4006	Weekly	Database known as “List of Medicinal Products for which Decisions Were Issued not to Allow Export from the Slovak Republic.”
Slovenia	Agency for Medicinal Products and Medical Devices (JAZMP), and Health Insurance Institute of Slovenia	http://www.jazmp.si/fileadmin/datoteke/seznami/SFE/Prisotnost/Seznam_44_HUM_prenehanja_motnje.pdf www.cbz.si	Irregular	Database known as “National Database of Medicines.” Only “yes/no” information on market availability.
Spain (1)	Spanish Agency of Medicines and Health Products (AEMPS), being part of the Spanish Ministry of Health	https://cima.aemps.es/cima/fichasTecnicas.do?metodo=buscarDesabastecidos	As required (whenever a shortage is detected)	Database known as “List of M with Supply Problems.” Database contains raw information on appearance of shortages, without secondary statistics.
Spain (2)	Center for Information on Medicines Supply (in Spanish: Centro de Información sobre el Suministro de Medicamentos; CISMED)	http://www.portalfarma.com/Profesionales/consejoinforma/Paginas/Infarma-2016-CISMED.aspx	Weekly	The CISMED produces reports including information on: Number of pharmacies which have requested but not received supply of a drug; The provinces where the drug is missing; Detailed information on drugs in a shortage situation and on equivalent medicines with lower, equal or higher prices; Drug's status: canceled, authorized, suspended, etc.; If it is a shortage informed by the AEMPS or not; Whether a drug in short supply is substitutable or not. Access to this database is limited to the CISMED's members.
Switzerland (1)	Federal Office of Public Health	https://www.bag.admin.ch/bag/de/home/themen/mensch-gesundheit/biomedizin-forschung/heilmittel/sicherheit-in-der-medikamentenversorgung.html?_organization=317	Weekly	Database known as “Security of Supply” (Versorgungssicherheit).
Switzerland (2)	Federal Office for national economic supply (FONES)	https://www.bwl.admin.ch/bwl/de/home.html)	Weekly	Database known as “Current Supply Bottlenecks” (Meldestelle Heilmittel—Aktuelle Versorgunsengpässe).
Switzerland (3)	Swissmedic (Swiss Agency for Therapeutic Products)	https://www.swissmedic.ch/swissmedic/en/home/humanarzneimittel/market-surveillance/out-of-stock.html	Weekly	Database known as “Out-of-stock Situation.” It provides information on periodical non-availability of therapeutically important medicines.
Switzerland (4)	Martinelli Consulting Switzerland	www.drugshortage.ch	Weekly	–

**Table 2:** Publicly available databases on drug shortages in countries outside of the area of the European Union and the European Free Trade Association.

**Country**	**Organization in charge of database**	**Access**	**Frequency of database updating**	**Notes**
Israel	Ministry of Health	www.health.gov.il	According to the need	Database known as “Warnings on Medications and Cosmetics Website; Notices of Medicines Marketing Interruptions.”
Kosovo	Kosovo Medicines Agency	www.akppm.com	–	–
Republic of Srpska, Bosnia and Herzegovina	The Agency for Medicinal Products and Medical Devices of Bosnia and Herzegovina (ALMBIH)	http://www.almbih.gov.ba/vijesti/	As soon as the ALMBIH is informed by the MAH about shortage of a given medicine	Information available under the section “News” on the ALMBIH internet site. Information on temporary shortages of medicines is provided to the ALMBIH by the appropriate MAH, including with reasons and expected date of availability.
Turkey	Turkish Medicines and Medical Devices Agency: TMMDA (in Turkish: Türkiye Ilaç Ve Tibbi Cihaz Kurumu; TİTCK)	http://www.titck.gov.tr/	Weekly	Only a part of the registry is made publicly available. TİTCK collects data on shortages by using the Pharmaceutical Track and Trace System to detect which medications are not available at the pharmacy level. If the stock of medication is below a certain level at the pharmacies (depending on the type of medication and whether it's an orphan or non-orphan status, etc.) it is labeled as passive. Similarly, a drug's statuses such as canceled, authorized, suspended, etc. are also labeled as passive. The list is integrated with e-prescription software that prohibits physicians from prescribing medicines which are unavailable. The list focuses on medicines used in out-patient settings.

In countries without national reporting systems, the evidence is dispersed and gathered independently by various stakeholders for their own purposes. For example, in Montenegro, this evidence exists fragmentarily at pharmaceutical companies or MAHs, wholesalers, pharmacies, Agency for Drugs and Medical Devices, MoH, and the Health Insurance Fund, among others. Information on shortages in Montenegro is not gathered systematically, but rather in situations when a more serious threat to continuity of care could be expected. In such cases it is the Agency for Drugs and Medical Devices and the MoH which are responsible for gathering this information. In those studied countries where reliable statistics exist, the assortments of medicines in short supply were generally well-recognized, and there was relatively accurate information on these. In Malta, the information on specific drug shortages was published only for publicly reimbursed medicines. Overall, the frequency of shortages of concrete medicines and their durations were described as “variable,” “rather not known precisely,” and “unpredictable.”

In almost all the countries, there exist formal obligations of pharmaceutical companies or the MAH to notify a certain organization or institution (“information hub”) in all or the majority of the following cases: (a) delayed or postponed commercialization of a medicinal product; (b) suspension, withdrawal, or lack of renewal of MA; (c) the predicted or sudden unavailability of a medicinal product due to other reasons; (d) the ceasing of reimbursement of a medicinal product; or (e) in other cases which could lead to drug shortages. The exemptions are Kosovo and Azerbaijan, but in the latter, although formally there is no such obligation specified in the legislation, such notifications are voluntary. Interestingly, in Norway the MAHs are not only obliged to notify the competent authorities but are expected to notify patient organizations on foreseen or existing drug shortages, although this is not required by law. In countries where reporting systems exist, at least one institution per country was included in the national reporting system, usually the national ministry responsible for health affairs or the agency responsible for the authorization of medicines. Moreover, in the majority of countries there was at least one additional institution (another hub) involved in gathering information, although in some countries (e.g., Belgium, Estonia, Poland) information was gathered by one institution but then further shared with others. The names of all the organizations involved in gathering information on drug shortages, including the main and additional ones which were identified in the studied countries, can be found in Tables 3, 4.

**Table 3:** Organizations involved in gathering information (“information hubs”) on drug shortages in countries of the European Union and the European Free Trade Association.

**Country**	**Main organization**	**Additional organizations**	**Notes**
Austria	Austrian Medicines and Medical Devices Agency (AGES MEA)	Main Association of Austrian Social Insurance Institutions (MAASSI) Pharmacists' Publishing House (http://warenverzeichnis.apoverlag.at/)	MAASSI does not publish information on shortages, but keeps track of shortages affecting reimbursed medicines, as drugs which are not available for longer periods will be delisted. The database of the Pharmacists' Publishing House requires payment to access.
Belgium	Federal Agency of Medicines and Health Products (FAMHP)	National Institute of Health and Disability Insurance (NIHDI; Dutch abbr. RIZIV; French abbr. INAMI); i.e., the Belgian national health insurance fund	FAMHP informs NIHDI of reported shortages of reimbursed medicines only.
Croatia	Croatian Health Insurance Fund	Croatian Agency for Medicinal Products and Medical Devices (HALMED)	Information gathered by HALMED is limited to products with ceased or not prolonged registration, stopped production, or importation (i.e., situations not necessarily considered shortages, according to some definitions).
Czech Republic	State Institute for Drug Control (SUKL)	Ministry of Health (Department of State)	Information gathered by both organizations is limited to suspended products (i.e., situations not necessarily considered shortages, according to some definitions).
Estonia	Estonian State Agency of Medicines (ESAM)	Estonian Health Insurance Fund (EHIF)	ESAM uses information derived from prescriptions, which is gathered by EHIF, to assess the need for products.
France	French Agency for Medicines Safety (ANSM)	Regional health agencies (Agences régionales de santé—ARS) National Council of the College of Pharmacists (Conseil national de l'ordre des pharmaciens—CNOP)	–
Greece	National Organization for Medicines (EOF)	Pharmacists' associations	The pharmacists' associations independently gather some information, but their data on shortages cannot be considered comprehensive or reliable (to the contrary of EOF's data).
Hungary	National Institute of Pharmacy and Nutrition	National Institute of Health Insurance Fund Management (NIHIFM) Wholesalers	NIHIFM needs to be notified only in the case of a drug shortage of reimbursed products.
Ireland	Health Products Regulatory Authority (HPRA)	Irish Pharmaceutical Union (IPU)—professional body of community pharmacists Wholesalers	The notification of HPRA is non-obligatory yet. The wholesalers keep track of current shortage situation to allow them to keep pharmacies informed. HPRA manage and approve “Batch Specific Requests.” HPRA may consider a batch-specific request application from a MAH in order to ensure the continued availability of a medicine on the Irish market.
Italy	Italian Medicines Agency (AIFA)	Regional health authorities	–
Latvia	State Agency of Medicines of Latvia (SAMLV)	Health Inspectorate National Health Service (NHS)	Information gathered by the NHS is limited to reimbursed products only.
Lithuania	State Medicines Control Agency	No other organizations	–
Malta	Medicines Intelligence and Access Unit, Malta Medicines Authority (MMA)	Central Procurement and Supplies Unit (CPSU), Ministry for Health (covers medicines supplied through the national public health system)	The Medicines Intelligence and Access Unit is responsible for managing a proactive and targeted approach to enhance medicines' intelligence and access. It supports the health care industry and all stakeholders in accessing medicinal products.
Norway	Norwegian Medicines Agency (NoMA)	Wholesalers	–
Poland	State Pharmaceutical Inspection	Ministry of Health	Full information on drug shortages is gathered by the State Pharmaceutical Inspection.
Portugal	Ministry of Health	INFARMED—National Authority of Medicines and Health Products ANF—National Association of Pharmacies CEFAR—Center for Health Research and Evaluation	–
Slovakia	The State Institute for Drug Control	Ministry of Health Health Insurance Fund The Slovak Chamber of Pharmacies	Information gathered by all Slovak organizations involved in gathering information on drug shortages is limited to reimbursed products only.
Slovenia	Agency for Medicinal Products and Medical Devices	Pharmacy Chamber of Slovenia Trade Chamber	–
Spain	Spanish Agency of Medicines and Health Products (AEMPS), being part of the Spanish Ministry of Health	Center for Information on Medicines Supply (in Spanish: Centro de Información sobre el Suministro de Medicamentos; CISMED) Databases run independently by governments of Autonomous Regions of Spain	Database of the governmental agency AEMPS contains publicly available but raw information on drug shortages, without any secondary analyses. Database of CISMED, created by pharmacists' professional organization—Collegiate Pharmaceutical Organization (in Spanish: Organización Farmacéutica Colegial) contains more complex information but is not disclosed to the public.
Switzerland	Swissmedic (Swiss Agency for Therapeutic Products) and Federal Office of National Economic Supply (FONES)	Manufacturers and other enterprises Wholesalers Professional organizations (e.g., hospital pharmacists' association GSASA) Hospitals, long-term care institutions for the elderly, etc	–

**Table 4:** Organizations involved in gathering information (“information hubs”) on drug shortages in countries outside of the area of the European Union and European Free Trade Association.

**Country**	**Main organization**	**Additional organizations**	**Notes**
Albania	National Agency of Drugs and Medical Devices	Health Insurance Fund	–
Azerbaijan	Center for Analytical Expertise of Medicines of the Ministry of Health,	Ministry of Economic Development	–
Israel	Ministry of Health	All four national Health Maintenance Organizations (HMO): Clalit Health Services, Maccabi Healthcare Services, Meuhedet Health Services, and Leumit Health Services	Access to the database of the Ministry of Health is open to the public. The other databases are shared only with certain employees of a given organization.
Kosovo	Kosovo Medicines Agency	Pharmaceutical Chamber of Kosovo Pharmaceutical Society of Kosovo	–
Montenegro	Montenegrin Agency for Drugs and Medical Devices	Ministry of Health Wholesalers	Information gathered by the Montenegrin Agency for Drugs and Medical Devices embraces all medicines marketed in Montenegro. The Ministry of Health gathers information only on medicines which are reimbursed by the Montenegrin Health Insurance Fund. All this information pertains to a rather more serious potential threat to continuity of care, since in the official regulation it is not explicitly stated that information on drug shortages has to be reported in Montenegro.
Republic of Srpska, Bosnia and Herzegovina (BIH)	Agency for Medicinal Products and Medical Devices of BIH	Ministry of Health and Social Welfare of the Republic of Srpska Health Insurance Fund Public Health Institute	Informing organizations which are listed as additional is non-obligatory for the MAH, nevertheless performed in practice. These institutions do not publish this information, but they are rather informed in order to take steps within their competence.
Serbia	Republic Health Insurance Fund (RFZO)	–	Information on medicines shortages is usually provided on a weekly basis by hospital pharmacists from the secondary and tertiary health care level. No feedback is provided by the RFZO on potential dates when shortages are expected to be resolved.
Turkey	Turkish Medicines and Medical Devices Agency (TİTCK)—Department of Economic Assessments and Drug Supply Management	Turkish Pharmacists Association (TPA) Social Security Agency (SSA)	The Department of Economic Assessments and Drug Supply Management is responsible for gathering information on shortages, managing prices of medications, contributing reimbursement decisions, and supplying unlicensed medications to patients and HTA. The MAH is responsible for notifying this department for shortages, possible stock problems, etc. Alerts can also be obtained from the public, physicians and pharmacists and wholesalers via various channels. There is a registry system for shortages; this registry is not publicly available, but it is shared with relevant organizations such as the SSA. This unit also undertakes measures to tackle and prevent shortages in collaboration with other health authorities, NGOs like TPA, and MAHs.

### “Bottom-up” initiatives

In some countries, bottom-up initiatives were undertaken such as working groups or informal networks targeted to tackle the problem of drug shortages; as well as guidelines, codes of conduct, good practices, or management plans related to drug shortages were also elaborated. These initiatives included establishing a working group at the hospital pharmacists' association and voluntary reporting of pharmaceutical companies to the pharmacists' publisher (Austria), or creating informal networks or committees tackling the problem of drug shortages (Czech Republic). Standard Operating Procedures for managing drug shortages were developed at the national institution responsible for drug registration in Estonia. In Belgium, a taskforce led by FAMHP and the National Institute of Health and Disability Insurance (NIHDI) was set up. The taskforce aims to prevent drug shortages through devising measures aimed at preventing drug shortages and minimizing the impact on patients through acting proactively on notifications of reported drug shortages and similar activities. In Belgium, the involvement of all umbrella organizations (such as the branded and generic industries, wholesalers, hospital pharmacies, community pharmacies, and insurance organizations) is considered essential to mitigate the problem of drug shortages. Surveillance and inspections were considered as important practices aimed at mitigating the problem of drug shortages in many of the studied countries.

In Hungary, a clearly outlined, step-by step practical guidance for health providers has been issued by the Professional College of Health Care (serving as an advisory body to the State Secretary of Health Care) on how to react in the case of drug shortages. It includes the following key areas: (a) preparing a strategic plan on tackling shortages; (b) contacting wholesalers and manufacturers to estimate the duration and severity of the drug shortage; (c) determining alternative treatments; (d) estimating the impact of the shortages on health and costs of treatment; (e) estimating the current stock of drugs affected by the potential shortage warning; (f) implementing official guidance on identifying and approving alternative treatments; (g) securing communication and patient safety; (h) collaborating with other external stakeholders; and (i) implementing prioritization among patients^12^.

In Norway, a hospital pharmacy working group was formed in Oslo to deal with drug shortages on behalf of all the hospital regions. They conduct weekly meetings, with representatives of the Norwegian Medicines Agency (NoMA) attending at least once a month or when needed. NoMA has also formed an internal “shortage team” consisting of pharmacists and physicians. This team assesses notifications and takes care of the majority of situations on an ongoing basis. In the case of more severe or complex shortage situations, the team might summon dedicated persons from several departments of NoMA to form an extended advisory group. The teamwork across the departments includes, for example, the Inspectorate responsible for “rapid alert” notifications and close contacts with NoMA representatives in international organizations, such as the Committee for Medicinal Products for Human Use or the Pharmacovigilance Risk Assessment Committee of the EMA, as well as the Co-ordination group for Mutual recognition and Decentralized procedures—human (CMDh), which originated at the network of the Heads of Medicines Agencies. This involvement has helped to ensure that situations occurring at EU level are handled in an efficient manner. In addition, NoMA has developed a wide and well-functioning national collaboration with the various stakeholders, in order to find appropriate country-wide solutions.

While in Norway the bottom-up initiative was started by hospital pharmacists, in Ireland the Irish Pharmaceutical Union (the professional body representing 95% of Irish community pharmacies) created an online platform providing up-to date information on drug shortages^13^. This was generally deemed to be a reliable source of information by the Irish pharmacists.

Numerous bottom-up initiatives and innovations within organizational frameworks aimed at coping with or preventing drug shortages have been developed in Spain. Due to the lack of precise information on shortages, the Center for Information on Medicines Supply (abbreviation in Spanish: CISMED) was created in 2014, as a bottom-up initiative of the Spanish pharmacists' professional organization (Collegiate Pharmaceutical Organization)^14^. It contains information on shortages that is voluntarily provided by pharmacists from across the whole country, as well as detailed reports and analyses on the shortages. The database of CISMED is run independently from the governmental database of the AEMPS^15^. Similarly, in Switzerland, in response to existing but unsatisfactory governmental efforts in the area of drug shortages prevention and information, a chief pharmacist at a regional hospital has successfully established an independent database, gathering alerts mainly from other hospital pharmacies in the country. Currently, this database receives by far the highest number of notifications as compared with the other Swiss drug shortages databases. For example, it receives ten times the number of notifications than the official governmental database run by the Federal Office of National Economic Supply (FONES), which gathers information only on disruptions in supply of medicines considered as “essential,” lasting for more than 14 days, and pertaining to the lack of presence of all registered doses and package sizes (i.e., shortages of selected doses and package sizes are not included as long as other doses and sizes are still available)^8^.

In Montenegro, some non-governmental organizations (NGO) and associations of patients with certain diseases (e.g., HIV-positive individuals) have started to tackle the problem of drug shortages, continually appealing through the mass media, social networks, and other ways of communication for potential solutions for drug deficiencies and shortages, with special attention paid to certain particularly sensitive groups of patients. In Serbia, occasional press publications appear, initiated by patients' organizations and aimed at raising awareness on drug shortages.

In Slovakia, in addition to the governmental organizations involved in gathering information on drug shortages, the Chamber of Pharmacies (Slovenská Lekárenská Komora—SLK) runs a drug shortages database and independently monitors and analyzes the situation in pharmacies. It actively informs pharmacies on actual drug shortages and cooperates with the State Institute for Drug Control and the MoH.

Initiatives in several countries have also included the imposition of quotas by wholesalers on sales to pharmacies. In Belgium and some other countries, the efforts to limit parallel exports have been initiated by the MAH via the imposition of quotas on orders of medicines, having a further impact (domino effect) at subsequent levels of trade. Paradoxically, quotas—although introduced in order to provide stable supplies of medicines in or at risk of shortages—contribute to the overall picture of shortages in many countries, especially impacting on health care facilities or patients whose access to medicines can be limited due to these quotas. Pharmaceutical companies in Lithuania sometimes create special schemes for their products, selling them only to selected pharmacies or having an agreement only with certain wholesalers, thus trying to protect their medicines from parallel export. Quotas in Poland were reported to be associated even with special verification of pharmacy orders by wholesalers requiring prescription scans from pharmacies.

### Other initiatives and organizational frameworks

In 2007, the Spanish Ministry of Health launched the “Coordinated Program of Control of Drug Supply” associated with a computer application called SEGUIMED (monitoring supply of medicines in the domestic market), which allows for making queries and reports on the status of distribution of medicines from when they leave the manufacturing plant until they are dispensed in pharmacies^16^. In this way, coordination among all the agents involved in the trade is facilitated, although the application was criticized by organizations representing major pharmaceutical distributors (FEDIFAR) as being obsolete^17^. In response to the communication of a supply problem, the AEMPS first assesses the possible impact that lack of supply could have on Spanish citizens. If this poses a threat to the citizens' health, it starts a series of measures aimed at resumption of supply and keeps health authorities of the Autonomous Communities informed. Besides administering a database on drug shortages, since 2009 the AEMPS also publishes information on its website on possible alternatives to the medicines in short supply. Furthermore, in some situations of shortages the AEMPS actively searches for medicines of foreign origin that could serve as alternatives to a nationally-produced medicine that is currently out of stock and it also authorizes its importation (Desabastecimientos de Medicamentos: Un Problema Sin Resolver, 2015). As mentioned, this is different to the CISMED initiative, which was developed to improve current knowledge.

A working group designed to assess severity of medicine shortages and propose actions was also set up by the National Organization for Medicines (EOF) in Greece. Moreover, one of the responsibilities of the Institution of Pharmaceutical Research and Technology, being a subsidiary of the EOF, is to cover demand when a given drug is in short supply, or its marketing has been discontinued. It includes searching for medicines on the international market outside of Greece, especially in case of medicines considered as unique or irreplaceable.

The State Medicines Agency in Latvia (SAMLV) has established a “Rapid alert working group,” taking decisions on necessary actions in cases of a detection of quality defects in medicinal products (including the appearance of counterfeit medicines)^18^.

Although the MAH is formally obliged to inform the State Medicines Control Agency (SMCA) in Lithuania, quite often this obligation is not fulfilled in practice. Therefore, the Head of the SMCA issued an order in 2012 aimed at combating drug shortages, according to which wholesalers send weekly reports to the SMCA on stock availability on the market. The SMCA experts analyze all the stocks which are reduced to a low level, highlighting medicinal products, places where potential shortages could appear, and checking if the reports of shortages were published by the MAH. When the information on the shortages is confirmed, the public is informed through the SMCA website both on the shortage of a particular medicinal product and the possible substitutes currently available on the market. Moreover, if a medicinal product of high importance (e.g., vaccine or reimbursed product) is in short supply, the SMCA immediately informs the MoH and the National Health Insurance Fund via an official note and attempts to find a solution. This includes, for example, finding and importing substitutes from other countries or negotiating higher production and supply levels with other retailers. Subsequently, the SMCA informs the public on the agreed solution, and the availability of the drug in short supply and its substitutes.

The MoH in Israel manages a drug shortages database, known as the “Notices of Medicines Marketing Interruptions.” They also send e-mail alerts to every registered health care provider in the case of some shortages, with an estimation on when they are supposed to be resolved. Israel's MoH has also published the procedure according to which every drug supplier must declare an expected drug shortage at least 3–6 months before it actually happens. In the case of serious problems arising from a shortage and fear for public health (for example if a drug fails to meet the Good Manufacturing Practice guidelines) the drug supplier must follow the European directive which defines this situation as urgent (Israel's Ministry of Health - Pharmaceutical Administration, 2011).

In Turkey, in order to prevent or cope with problem of drug shortages, the national medicines agency TİTCK (responsible for, among others, taking the necessary precautions to ensure that vital medications, medical devices, and health products are constantly available on the market) established the Department of Economic Assessments and Drug Supply Management in 2012^19^. This department is responsible for registering drug shortages, managing stocks, taking necessary actions to solve shortages-related problems or preventing them in the future, and notifying the relevant stakeholders such as the Turkish Pharmacists Association. It is also responsible for managing the prices of medications (so they can intervene if the shortage is dependent on the price), supplying unlicensed medications from other countries if necessary, and giving priority to certain medications when licensing them.

In Azerbaijan, in order to gather information on drug shortages directly from patients, a hotline was established at the Center for Analytical Expertise of Medicines of the Ministry of Health. A special commission was also set up at the MoH, one of the tasks of which is to monitor drug shortages. All the information on the shortages of medicines is then transferred to the Ministry of Economic Development.

### Influencing trade rules (without a direct impact on arbitrage/parallel trade)

Measures aiming to mitigate or prevent drug shortages in the various countries involved in this study, influencing trade rules but not having a direct impact on arbitrage or parallel trade, were associated mainly (but not exhaustively) with the approach to public service obligations (PSOs) and the possibility to prescribe medicines which were not registered in a particular country.

PSOs associated with supplying medicines to cover patients' needs, so as not to compromise patient care, exist in the majority of studied countries. However EU legislation, and the way this is transposed into the national legislation of EU countries, opens a doorway to increased transparency. According to Article 81 of Directive 2001/83: “The holder of a marketing authorization for a medicinal product and the distributors of the said medicinal product actually placed on the market of a Member State shall, within the limits of their responsibilities, ensure appropriate and continued supplies of that medicinal product to pharmacies and persons authorized to supply medicinal products so that the needs of patients in the Member State in question are covered^20^.” Interestingly, the PSOs have been implemented also in several non-EU states. For example, in Turkey the PSO is stated as follows: “MAHs are responsible to provide market availability for their licensed products” and “MA can be canceled if the MAHs cannot manage to supply their products to the market^19^.”

In the majority of studied countries, the PSO could embrace all registered products, although in Poland it is more strongly expressed or followed more carefully in the case of reimbursed products. In Croatia, Italy, Slovakia, and Serbia, it is limited to publicly reimbursed products only. In Albania, the PSO is specific to products which can be reimbursed or bought directly by the state, as part of specific reimbursement lists, or products included into public hospitals drug lists. As soon as the PSOs are in place, they are formulated within a given country's legislation, with the exception of Estonia. The pharmaceutical companies in Poland are obliged to declare a certain volume of sales for each year of the reimbursement decision's duration, and they are expected to pay fines in the case of shortages. In Montenegro, the PSO pertains to providing continuous supply of medicines rather than covering the needs of the country's population, and in the case of reimbursed medicines—to providing their availability to the insured persons. The PSO in Slovakia is associated with timeframes within which the MAHs and wholesalers are obliged to deliver medicines included in the national reimbursement list–24 and 48 h, respectively, from receiving an order for a given medicine. On the other hand, a pharmacy is obliged to accept delivery of the ordered medicine from the wholesaler within 24 h from placing their order (Slovak Parliament, 2011). We identified a very special approach to PSO in Switzerland, where 60 medicines considered by health authorities as essential and of the highest importance to public health (regardless of their reimbursement status) must to be stockpiled by manufacturers, but the conditions are negotiated and set in agreements between them and FONES^8^. PSOs do not exist in Azerbaijan and Kosovo.

Typically in smaller countries, local authorities can find it difficult to enforce PSOs. The authorities try to motivate pharmaceutical companies to register products rather than making them delist these products. Consequently, in some cases enforcement of PSOs can have a negative effect on availability since it can backfire e.g., if a product is delisted, it cannot even be parallel imported. Countries with small pharmaceutical markets can in some cases provide solutions aimed at mitigating possible problems associated with supplying them with medicines and facilitating trade. For example, in Albania, an additional adhesive label with information in Albanian language can be placed onto the original outer packaging. This is the case in situations including the supply of first alternative drug or drugs for hospital use, where the quantity does not exceed 4 months' supply, or when a given drug constitutes the only alternative treatment available on the market. This is to avoid delays in the market supply^21^.

One of the possible remedies in the case of drug shortages is the possibility for physicians to prescribe an unlicensed medicine if a registered product is unavailable on the market. In the EU Member States, this refers to the possibility of use of Article 5 of Directive 2001/83/EC, and the prescribing of medicinal products without a MA under Article 5 still requires some form of authorization by the national authorities^20^. Such alternative options are formally available to physicians in all studied countries. Obtaining a special permit or exemption from the responsible state authorities is usually necessary in such cases. In Hungary, there are several administrative obstacles (including the special individual approval of the National Institute for Pharmacy and Nutrition) to prescribing an unlicensed medicine, although such an option is formally allowed. In the case of drug shortages with expected severe consequences, a so-called “contingent approval” to import a larger amount of non-registered products may be given to wholesalers to bypass the bureaucratic impediments of individual approval^7^. In Ireland, the granting of a temporary authorization for a batch of a medicine which is not registered but necessary for patients, is based on a “batch specific request,” reviewed by the Health Products Regulatory Authority (HPRA) (Health Products Regulatory Authority, 2017). Such applications may be appropriate when a medicine in full compliance with its registered MA dossier is temporarily unavailable or where action is proposed to bring a batch into compliance with the registered details.

Similarly, the possibility for pharmacies or wholesalers to import unlicensed medicines is available in a majority of European countries. In France, a medicinal product must be in any case authorized by the French Agency for Medicines Safety (ANSM) to be imported either by a standard MA, by a temporary use authorization, or a specific authorization. Importation authorizations are restrictive in France; they are delivered for a maximum of 1 year and for a fixed volume. Belgian pharmacies can import such medications only in the case of a shortage of an equivalent product being confirmed by the FAMHP and a physician's declaration is provided stating that a medicine is actually necessary for a patient. In Switzerland, the importation permits for unlicensed medicines are issued by Swissmedic, the national drug registration agency.

In BIH, the MoH of the Republic of Srpska or the MoH of the Federation of BIH (the two political entities that compose BIH) may authorize the import of a medicine without a MA issued by the Agency for Medicinal Products and Medical Devices of BIH. It can happen only in cases of an urgent, medically justified need of a particular patient or the need of using limited quantities of medicines which are necessary for the population's health protection. In both situations, the application must be issued by the health facility. The import permit given to wholesaler may be authorized only in case of unavailability of a medicine with MA (The Parliamentary Assembly of Bosnia Herzegovina, 2008).

In Montenegro, the possibility to prescribe an unlicensed medicine plays an extremely important role. Due to a small market size, the country is encountering a lack of interest in the registration procedure and, as a result, ~20% of medicines are being placed on the market via the special import procedure, based on the Law on medicines (Government of Montenegro - Ministry of Health, 2011). In such cases, the Agency for Drugs and Medical Devices grants an import license for non-registered medicine(s) to a wholesaler, which will import that medicine(s) and deliver it to a given health institution or pharmacy. This is important for specific patients or groups of patients who need such particular medicines and cannot be adequately treated with medicines registered on the Montenegrin market. Moreover, importing medicines from countries with very similar official languages as Montenegrin (Serbia, Croatia, Bosnia and Herzegovina), following approval of the Montenegrin Agency for Drugs and Medical Devices, which is based on registration issued in these countries, facilitates supplying the national market and assists in preventing drug shortages. This simplified approach becomes invalid when differences exist in the documentation between Montenegro and these countries and the labeling has to be clearly specific for Montenegro.

In order to cope with severe supply problems and shortages of medicines in Kosovo, several changes were made to the legislation to allow for the import of products without MA. As a result, if there are no products identified by their International Non-proprietary Name and pharmaceutical form and which have MA issued in Kosovo, then wholesalers can import them from any of the EU Member States. This was implemented to ensure that the appropriate national authority has already considered the quality issues. A similar procedure is followed when a given product has obtained MA but is not present on the market—in such a case the national Agency can suspend MA for a given period and permit the import.

Several innovative practices have originated in Malta, where the specifics of this small-sized national pharmaceutical market prompted the authorities to use a number of measures available under EU legislation. This especially included implementation of Article 126a of Directive 2001/83/EC, enabling authorizing the placing of a medicinal product with a MA in another EU Member State on the Maltese market in the absence of a MA or of a pending application in Malta^20^. There have also been other measures implemented, including that medicinal products could be placed on the market in any one of the official languages of Malta (i.e., Maltese or English), or accepting the possibility of having joint packs with other larger English-speaking countries, mainly the UK and/or Ireland (Vella Bonanno and Gavril, 2011). The issuing of some authorizations for distribution of unauthorized medicines based on Article 126a of the Directive 2001/83/EC was also reported from Latvia^22^.

Last but not least, in Hungary, for some key products the National Institute for Pharmacy and Nutrition asks for stock reports from wholesalers, manufacturers, and hospitals to prevent shortages. In the case of generics, where internal reference pricing is applied, should the continuous supply of the lowest price (reference) product not be further guaranteed, the preferred status of that product may be removed. In such a case, other drugs with the lowest daily treatment cost may be awarded the status of preferred (reference) products (Hungarian Minister of Health, 2004).

The summary information on measures influencing trade rules concerning medicines can be found in Tables 5, 6.

**Table 5:** The summary information on measures influencing trade rules of the European Union and the European Free Trade Association.

**Country**	**Public service obligations:** **A—pertaining to all medicines** **R—limited to reimbursed medicines only** **N—nonexistent**	**Possibility to prescribe non-registered medicine:** **Y—allowed** **N—not allowed**	**Limitation of arbitrage/parallel export:** **N—not possible** **O—possible but not executed** **Y—possible and executed**
Austria	A	Y	N
Belgium	A	Y	O
Croatia	R	Y	N
Czech Republic	A	Y	Y
Estonia	A	Y	Y
France	A	Y	Y
Greece	A	Y	Y
Hungary	A	Y	O
Ireland	A	Y	N
Italy	R	Y	O
Latvia	A	Y	N
Lithuania	A	Y	N
Malta	A	Y	O
Norway	A	Y	O
Poland	A	Y	Y
Portugal	A	Y	Y
Slovakia	R	Y	Y
Slovenia	A	Y	N
Spain	A	Y	Y
Switzerland	A	Y	O

**Table 6:** The summary information on measures influencing trade rules in countries outside of the area of the European Union and European Free Trade Association.

**Country**	**Public service obligations:** **A—pertaining to all medicines** **R—limited to reimbursed medicines only** **N—non-existent**	**Possibility to prescribe non-registered medicine:** **Y—allowed** **N—not allowed**	**Limitation of arbitrage/parallel export:** **N—not possible** **O—possible but not executed** **Y—possible and executed**
Albania	A	Y	N
Azerbaijan	N	Y	N
Israel	A	Y	N
Kosovo	N	Y	Y
Montenegro	A	Y	N
Republic of Srpska (BIH)	A	Y	N
Serbia	R	Y	N
Turkey	A	Y	Y

### Influencing medicines' trade rules in response to arbitrage/parallel trade

Since one of the possible reasons for drug shortages can be arbitrage or, as it is officially referred to in the case of EU countries, parallel export of medicines from markets where they are relatively cheaper to markets where their prices are higher, measures were taken in some countries to limit parallel export. EU legislation allows for limiting parallel export if public health in a Member State is endangered. However, not all EU countries have introduced specific measures into their legislative systems. In the Czech Republic, for example, the legislative changes were approved and effective recently, in April 2017. Nevertheless, these legislative changes mainly reflect long-term practice in this respect, in the Czech Republic (e.g., the competence of the MoH to communicate with exporters). In Italy, the formal possibilities to limit parallel export temporally do exist through issuing of the MoH's decisions, although they have not been executed yet. Generally, the forms of limiting arbitrage or parallel trade appear to be highly differentiated (Tables 5, 6). In some of the studied countries, they are made at the highest official level—through the issuing of banning decisions by, for instance, the MoH in Poland, the National Medicines Agency in Estonia, or jointly by the National Medicines Agency and the Ministry of Economics in Turkey. The possibility to limit parallel trade of medicines in Slovenia is executed indirectly through the inspection of pharmacies.

Since parallel export is considered by the Polish MoH as an important issue, the crucial top-down change that was introduced in May 2015 established a list of medicinal products, food products for special dietary use, and medical devices vulnerable to lack of availability on the territory of the Republic of Poland^23^. Any wholesaler aiming to export a listed product from Poland is obliged to obtain permission from the Main Pharmaceutical Inspectorate. This legal change appreciably reduced the problem of drug shortages, although the illegal re-exportation (reversal of trade chain) of medicines still remains a serious problem. It is associated with illegal practices of pharmaceutical entrepreneurs, pertaining to prohibited trading of medicines from pharmacies or health care facilities to wholesalers and then exporting them. In order to eliminate illegal practices, all Polish pharmaceutical market entrepreneurs will have to submit detailed information on traded medicines into a special system (the Integrated System of Monitoring Trade of Medicinal Products) starting from 2018^24^. The Polish health authorities will be able to track every single package of a medicinal product on the Polish market. Except for the additional benefits expected from launching this system (e.g., increased pharmacovigilance and safety of medicines use), it should diminish the scale of the reversed chain of trade. Interestingly, a specialized system for tracking medications throughout the distribution chain (from manufacturing process to patients), the Pharmaceutical Track and Trace System (İTS), has also been developed in Turkey. When excessive export of medicines occurs, the İTS allows to track it as well^25^.

Since relatively low prices of medicines in Poland increase the probability of exporting them, recently the Polish MoH also had the controversial idea of increasing official (nominal) prices in order to bring them closer to levels from more affluent Western European countries, where they are often much more expensive. At the same time, effective prices were supposed to stay at their current levels through using confidential risk sharing arrangements (instruments). Such changes were only possible in the case of reimbursed drugs. Nevertheless, the MoH canceled the implementation of this initiative. In addition, in France, as in Poland, it is prohibited by law to export medicines of major therapeutic value in case of a drug shortage. In Greece, prohibition of parallel exporting was imposed initially on vaccines and then also for other medicinal products.

In Spain, several measures to prevent excessive export of medicines have been developed. Development of distribution activity to other countries, undertaken by pharmacies and other entrepreneurs, concerning medicines with supply problems which could have an impact on health care in Spain, has been considered to be a very serious problem since 2015 (King of Spain, 2015). Compliance with Good Distribution Practices has been strongly required at least since 2013, including measures aimed at preventing reverse distribution, as well as obligatory notification of suspected drug diversions^26^. The formally sanctioned priority in medicines distribution in Spain is supplying the national pharmacies. MAHs and wholesalers should ensure, within the limits of their responsibility and within the agreed delivery times, an adequate and continuous supply of medicines to Spanish pharmacies so that the needs of the patients are covered. In cases where there are problems of supply of medicines whose shortages may have an impact on health care, the AEMPS will play a key role^26^. This pertains to situations when: (a) it is a drug that, because of its active ingredient, dosage or route of administration, is the only one registered in Spain for a particular disease and its absence generates a therapeutic gap; or (b) it is a medicinal product whose absence implies a modification of the prescription. Under these circumstances, the AEMPS should adopt the measures it deems necessary to resolve this situation and may limit the export of medicines based on the need for protection of public health, in collaboration with the competent authorities of the Autonomous Communities. Autonomous Communities have developed their numerous supplementary rules. Exporting medicines on which an export ban was imposed due to shortages and threat of the danger to public health may result in penalties ranging from EUR 90,000 to EUR 1 million^27^.

Important measures in Slovakia, primarily aimed at preventing parallel export of medicines reimbursed in the country, have come into effect in January 2017. These measures were also aimed at the removal of accusations for which the European Commission brought proceedings against the Slovak Republic (the first banning of re-exporting medicines started in 2012). The new rules ensure that distributors are no longer able to export drugs covered by public health insurance, since such exports are only permitted with written authorization/power of attorney of the MAH. Both distributors and producers are obliged to supply medicines only to local pharmacies. When attempting to export medicines, distributors must give notice at least 7 days beforehand. Local pharmacies are also currently prohibited from re-selling drugs to any distributors except authorized wholesalers, since in the past the drugs purchased by distributors from pharmacies were often subsequently exported (Slovak Parliament, 2011; Pfeffer and Mozolová, 2018). These recent measures taken in Slovakia have effectively fulfilled their main objective of preventing the re-export of medicines. On the other hand, generic pharmaceutical companies started withdrawing hundreds of generic medicines due to their fear of huge fines. As mentioned above, all drugs from the national reimbursement list have to be available within a very short time from order. In addition, sanctions in the case of breaching the new law include fines of between EUR 5,000 and EUR 1 million, as well as the possibility of distributors or pharmacies losing their operating licenses.

## Discussion

Drug shortages emerged as a universal problem in all the countries studied, regardless of their geographical location, level of economic development, or type of health care system. Despite being such a common issue, they are rarely defined in a precise way within the studied countries' legislations. Much more frequently, they were defined indirectly or not defined at all. This finding is consistent with the previous study, according to which 26 very different definitions for drug shortages were identified globally in 2015 across a wide array of sources, including: national laws, governmental and professional organizations, and scientific papers (De Weerdt et al., 2015a), as well as other recent studies (De Weerdt et al., 2017b). At EU level, the concept of drug shortages on which the EMA focuses its interest has been expressed through the description of shortages qualifying for inclusion into the EMA's medicine shortages catalog (cit.: “… medicine shortages that affect or are likely to affect more than one European Union (EU) Member State, where the European Medicines Agency has assessed the shortage and provided recommendations to patients and healthcare professionals across the EU”) (European Medicines Agency, 2017).

The definitions of drug shortages tend to be remarkably differentiated, depending on whether they pertain to supply problems or actual drug shortages, permanent or temporal discontinuations, typology of affected disease classes, and time frame. Generally, drug shortages can be expressed in four different ways, according to their focus on either the demand or supply side, and the way they impact either drug delivery or availability to patients (De Weerdt et al., 2015a). Besides, the notion of drug shortages depends very much on stakeholder's position within the medicines supply chain: e.g., a manufacturer, wholesaler, hospital or community pharmacist have varying perspectives on what constitutes a shortage (Kavanagh, 2017).

Lack of any definition adopted within a national health care system, as well as diversity of definitions in the same system, or incomparability and inconsistency of definitions across systems or countries, can negatively affect reporting, communication, and comparative analyses of the problem of drug shortages, including shortages' scale and effects. Trying to impose any single “ideal” definition of drug shortages, officially and internationally replacing the already existing expressions or descriptions for the studied phenomenon, is almost impossible and unnecessary. However, a set of very precisely worded definitions of major problems should be established, comprehensively defining what we understand to be drug shortages. This set of definitions should subsequently be proposed to the international community of pharmaceutical policy researchers and professionals, then discussed, mutually agreed, and recommended for wide usage—at least for the purposes of reporting these drug shortages, occurring locally or nationally, the reports of which are made publicly available. In this way, international cooperation on solving or mitigating the problem of drug shortages would be facilitated and strengthened.

The need for developing the commonly agreed terminology (Bogaert et al., 2015) and obtaining a uniform definition for a drug shortage (De Weerdt et al., 2015a) were reported in the previous studies as well. Just recently, two draft definitions of drug shortages were prepared under the auspices of the WHO (Heiskanen et al., 2017). According to the WHO, on the supply side: “A “shortage” occurs when the supply of medicines, health products and vaccines identified as essential by the health system is considered to be insufficient to meet public health and patient needs. However, this definition refers only to products that have already been approved and marketed, in order to avoid conflicts with research and development agendas.” (World Health Organization, 2016). On the demand side: “A “shortage” will occur when demand exceeds supply at any point in the supply chain and may ultimately create a “stockout” at the point of appropriate service delivery to the patient if the cause of the shortage cannot be resolved in a timely manner relative to the clinical needs of the patient” (World Health Organization, 2016). Hopefully, the efforts of the WHO, pharmaceutical policy researchers and professionals, and the EMA will finally result in establishing a set of definitions of drug shortages, which would be suitable for adoption at the EU level and globally.

The occurrence of drug shortages in recent years was rather constant and their dynamics tended to increase—both features being quite differentiated among the studied countries. However, due to the existing limitations and variabilities of the reporting systems, more advanced comparisons and conclusions are difficult. These could be facilitated not only by using the commonly agreed definition of the studied problem and nomenclature for its description, but also having a standardized methodology of reporting drug shortages. It is plausible that information on drug shortages is being made publicly available in many of the studied countries. However, as expected, we found that these databases are published in local languages only, which does not help in performing systematic, periodically repeated in-depth analyses. Having at least a basic, explanatory description of the structure of each publicly available database on drug shortages—translated into the widely-used English language—would facilitate further research. While researching the problem of drug shortages, it is also important to take into consideration a baseline level of analysis and the historical circumstances influencing the development of a given health care system. At the EU level, involvement of the EMA could have a crucial role, while the WHO could facilitate efforts at the global level. The development of initiatives and networks of professionals and academics, like COST Action CA 15105 “European Medicines Shortages Research Network—addressing supply problems to patients (Medicines Shortages),” encouraging systematic sharing of information and research, seems to be promising and points the way forward^28^.

Providing public access or removing obstacles in accessing drug shortages databases for interested stakeholders, seem to be the critically important factors which facilitate research on drug shortages. On the other hand, the dispersion of organizations responsible for gathering information on drug shortages in the same country (e.g., Switzerland) can possibly lead to a multiplication of efforts or confusion in interpreting the results of analyses—especially when the methodologies of gathering information are differentiated among such organizations. This provides an additional rationale not only for developing agreed and commonly understood sets of definitions, but also for methodologies for reporting and analyzing drug shortages within countries. The obligations of the pharmaceutical industry to notify certain information hubs in cases leading to drug shortages, existing in the majority of the studied countries, are crucial with respect to maintaining efficient information systems.

The role of civil society appears to be important in coping with the problem of drug shortages. Generally, the multitude and diversity of organizational frameworks and bottom-up initiatives, independently developed in the studied countries, seem to be one of the most inspiring and optimistic findings. We identified innovative solutions, developed both by governmental and provincial institutions, as well as grass-roots initiatives originating at professional organizations or health care providers. Interestingly, comparison of ingenuity of initiatives developed in the EU vs. non-EU countries does not seem to reveal major differences. It has recently been argued that there may be a potential link between drug shortages and increasing drug prices, especially in the USA, where health care organizations are experiencing unexpected price increases for generic or older products (Fox and Tyler, 2017). For instance, more than 300 generics in the US experienced price rises of 100% or more between 2010 and 2015 (Office UGA, 2016). The factors that can increase the risk of drug shortages are the same factors that can also augment the risk of raising drug prices. On the other hand, the same strategies can be used to manage drug shortages and minimize the impact of unexpected price increases. Based on this, the clinician checklist was proposed that includes four steps: (a) assessment of clinical use; (b) check of purchasing and inventory practices; (c) evaluation of operational opportunities; and (d) communication about the issues (Fox and Tyler, 2017). In Canada, the best practices guidelines for the prevention, notification, and management of drug shortages have also been developed by the pharmaceutical generic industry association. These guidelines are focused on ways of notification of stakeholders, communication strategies and management of shortages^29^. Regardless of the health care system's sector, there are a few common core values that are necessary when handling drug shortages. These include: establishing effective communication channels, creating ongoing collaborative relationships among all the stakeholders (including manufacturers and professional organizations) and also internally (within an organization), as well as having keen foresight and acting proactively, as opposed to just reactively, to a given situation (Dill and Ahn, 2014).

The public service obligation imposed on suppliers of medicines can potentially play an important role in securing appropriate access to drugs and preventing their shortages. However, we found that PSOs are often limited to reimbursed medicines only, and difficult to execute in practice in the studied countries. The approach to and interpretation of a PSO was found to be differentiated, both among the EU and non-EU/EFTA countries. The standpoint of the European Association of Pharmaceutical Full-line Wholesalers (Groupement International de la Répartition Pharmaceutique—GIRP) is that provisions of the PSO for persons active in the distribution of medicines should be compulsory in all Member States of the EU^30^. PSOs imposed on full-line wholesalers are aimed to secure the patients with several benefits. They can receive their medicines in a timely and safe manner from reliable and certified sources, which offer an adequate range of permanently guaranteed products to them. Besides, due to the PSO, the medicines which patients receive meet the requirements of a specific geographical area, and are delivered according to the supplies requested within a short timeframe. Consequently, according to the GIRP, the European framework providing for PSOs for pharmaceutical full-line wholesalers would enhance and strengthen the guarantee to patients in obtaining their medicines^30^. The European Association of Pharmaceutical Industries and Associations admits that strict adherence to regulatory obligations is essential to the integrity and reliability of the overall supply chain, while PSO should be interpreted and enforced in a proactive manner which prioritizes patient safety (European Association of Pharmaceutical Industries Associations, 2014). PSO must be warranted on grounds of public health protection, and proportionate in relation to the objective of such protection^20^.

The possibility to prescribe unlicensed medicines in the case of non-availability of other medicines, is an important public health safety measure, especially in smaller countries where the size of the pharmaceutical market and the number of potential users are not particularly attractive to the pharmaceutical companies. Generally, in smaller countries we found several interesting initiatives to cope with the problem of drug shortages, such as multi-language labeling or simplified labeling in some limited cases.

Parallel trade or arbitrage on a pharmaceutical market is a very complicated issue. In the EU, it is founded on the cardinal rule of free trade of goods, while on the other hand the Member States are responsible for their independent, national, or even local pharmaceutical policies (including pricing and reimbursement). Being supported by these two pillars, parallel trade in the EU has very differentiated implications. For example, its impact on patients' expenditures on medicines as it is very much dependent on the rules of copayment (Kanavos et al., 2004, 2006; Glynn, 2006). Parallel trade impacts the pharmaceutical trade as well as health care systems, and patients in numerous and sometimes unexpected ways; nevertheless, it seems that the EU is simply destined to function with parallel trade of medicines.

As it was revealed already in 2004, in the report on the economic impact of pharmaceutical parallel trade in EU Member States, the price spread between an exporting and importing country was a key factor in determining the potential for parallel trade, with the market size of the importing country determining its extent (Kanavos et al., 2004). The direct financial effects were assessed in six countries, and 19 products were analyzed in that report. Patients were found not to be benefitting directly from parallel trade, while the financial benefits accrued to health insurance organizations or pharmacists were, at best, modest. Significant benefits were identified among parallel importers, while manufacturers (particularly of products under patent) incurred a significant loss of business in the destination countries. The hypotheses of price convergence across (importing and exporting) countries, as well as price competition and a downward price spiral within importing countries, were rejected. Taking into account that some exporting countries may experience product shortages, it was concluded in that report that the static welfare effect of parallel trade was at best neutral (Kanavos et al., 2004). Our findings indicate that parallel trade is perceived as a major factor contributing to drug shortages and that an increasing number of states are implementing legal solutions aimed at controlling parallel export from their national territories. We found increasing efforts to curb parallel export in cases when it hampers the secure provision of medicines to citizens and more generally—public health. It should be stressed that the real impact of drug shortages (considering their diversity and assortment) on public health is still not known precisely in many countries. While the significance of life-threatening shortages of certain medicinal products is indisputable, in many cases the share of life-threatening shortages among all the shortages often remains unknown or unquantified.

There appears to be an insufficient role and relatively poor engagement of the EU bodies in coping with the problem of drug shortages. The need for a greater involvement of the EU institutions has been already expressed in Ireland. In its 2016–2020 strategic plan, the Irish HPRA suggested that more effective solutions will be possible through EU co-operation on issues such as medicines shortages (Health Products Regulatory Authority, 2016). The role and engagement of relevant EU bodies seems to be especially important nowadays in light of the differentiated, and just recovering from the lower levels, image of the EU among citizens of the Member States (40% of total positive answers in 2017 vs. 52% in 2007, according to the Eurobarometer periodic survey), along with the only just recovering trust toward EU institutions (42% tending to trust in 2017 vs. 57% in 2007) (European Commission Standard Eurobarometer, 2017). The stronger involvement of EU institutions in coping with the problem of drug shortages could be an appreciable opportunity to stand by the citizens of virtually all Member States, since the problem of drug shortages is vital to all citizens, patients, and their families across the whole of the European Union.

It should be noted that this study is not without limitations. The large number of countries and the broad scope of the issues investigated prohibited the authors from conducting an in-depth analysis of each aspect of drug shortages. The choice of survey respondents mainly from national health authorities and the policy area, and not taking the perspective of other stakeholders, such as manufacturers, wholesalers, or pharmacists with knowledge of the problems and potential interventions, could give a one-sided perspective and potentially bias the results. Furthermore, utilizing the personal knowledge of survey participants, even expanded by using the published sources as well, could be a further potential limitation of the study. However, we typically took the health authority perspective as they are the ultimate payers. The methodology applied in this study and character of the gathered material also did not allow for detailed quantitative assessment and comparative analysis of the extent of drug shortages, the assortment of products with shortages and the changes in shortages of certain medicines over time. Consequently, appropriateness and effectiveness of potential interventions, which could be different for unpredictable and predictable shortages, could not be fully assessed based on the gathered material. More detailed characteristics of drug shortages, analyses of information systems and their role in coping with shortages, detailed descriptions of initiatives, and organizational frameworks preventing shortages, as well as analyzing how interventions into trade rules, including also the approach to parallel trade or arbitrage, should be further studied and internationally compared and evaluated to provide potential ways forward. However, the authors believe that this study provides a very comprehensive overall picture of the drug shortages landscape in a broad range of countries and lays out directions for possible future research. Considering the still limited evidence on drug shortages, the authors have focused on including various European and Western Asian countries in order to provide a broad overview in a single study. This has inevitably raised the issue of comparability of national or local experiences. However, the authors believe that their inclusive approach has resulted in more advantages than constraints in the interpretation and drawing up of lessons for the future.

## Conclusions

Very different definitions of drug shortages exist in a small number of countries, while in the majority, no definition was adopted at all. There is an urgent need to develop an agreed set of definitions of drug shortages, as well as methodologies for drug shortages' evaluation and monitoring. They should be proposed, discussed among pharmaceutical policy researchers and professionals, and agreed and adopted for use on an international scale. There is a role for the WHO and the EMA to further engage and take a lead.

The characteristics of drug shortages, including their duration, frequency, dynamics and assortment of products are variable and sometimes difficult to assess. Although drug shortages have been very often, or always, present in the majority of studied countries, they are now universally perceived as a public health problem. There usually exist information hubs in the studied countries gathering evidence on drug shortages. Sometimes the content of the databases is limited to reimbursed products only, or not fully disclosed to the public. Providing public access to information on drug shortages, to the maximum extent possible, is a prerequisite for performing more advanced studies on the problem and identifying its solutions.

An optimistic finding of this study was the identification of numerous bottom-up initiatives and organizational frameworks aimed at preventing or mitigating drug shortages. The efforts of governments or state health authorities are often supplemented or excelled by independent actions originating at pharmacies, professional organizations or associations, as well as lower-level local health authorities. However, the need for bottom up approaches could also be a symptom of the national regulatory bodies not adequately addressing the issue. The experiences and lessons drawn from these initiatives should be carefully evaluated, monitored, and presented to the wider international audience for potential development beyond the local country. This paper is a start in this process.

The various forms of influencing trade rules, as the strategies for coping with the problem of drug shortages, are being implemented in the studied countries. Imposing PSO on pharmaceutical manufacturers, wholesalers, or pharmacies is rather difficult, especially in smaller countries. Providing the formal possibility to prescribe (and trade) unlicensed medicines, when necessary, is a widespread measure to prevent serious crises in drug supply chains and health care systems. Influencing rules of parallel trade or arbitrage, through imposing bans on exporting medicines in the case of threat to public health evoked by drug shortages, is increasingly gaining in popularity, and will continue.

The authors' intention is that this paper provides a platform to tackle the increasingly important public health issue of drug shortages, and will be taken forward by them.

## Author contributions

TB coordinated the project and conceived the conception and design of the study, including preparation of protocol, and questionnaire. TB carried out the data management and performed analysis and interpretation of data for the work. BG revised critically the questionnaire and contributed to critical analysis and interpretation of data for work. The following authors contributed in acquisition of data from respective states: AM, IHo (Albania), AB (Austria), VA (Azerbaijan), EDW, SS, IHu (Belgium), VM-P (Rep. of Srpska, Bosnia and Herzegovina), LS-B (Croatia), JS, RS (Czech Republic), KM, OL (Estonia), M-CL (France), LT, DM, MG (Greece), AI, BH (Hungary), VH (Ireland), MD, DS (Israel), RJ (Italy), AJ (Kosovo), EG (Latvia), JG (Lithuania), PV (Malta), ND-K, BA, ZB (Montenegro), TH (Norway), TB, EW (Poland), JdM (Portugal), NM, DR (Serbia), TT (Slovakia), JF, SP (Slovenia), IdMB (Spain), HJ (Switzerland), EUG, AA (Turkey). TB drafted the manuscript. All authors read and revised critically the subsequent versions of manuscript for important intellectual content. All authors finally approved the version to be published and agreed to be accountable for all aspects of the work in ensuring that questions related to the accuracy or integrity of any part of the work are appropriately investigated and resolved. TB is the corresponding author and the guarantor.

### Conflict of interest statement

SS, IHu and EDW have received funding from Research Foundation Flanders (FWO) and from TEVA to conduct academic research on drug shortages. MG has received research funding from Novartis, Glaxo-Smith-Kline, Boehringer-Ingelheim, Bayer, Merck, TEVA, ELPEN, and honoraria from Astra-Zeneca, Novartis, Chiesi, ELPEN, Pharmaten, Boehringer Ingelheim, Glaxo-Smith-Kline and Menarini. The other authors declare that the research was conducted in the absence of any commercial or financial relationships that could be construed as a potential conflict of interest.

However, some of them work directly for national health authorities or are advisers to them, are academics or independent researchers also working with national and regional health authorities and others to improve the access to medicines. The content of this manuscript, as well as all conclusions, views and opinions expressed therein, are those of the authors and may not necessarily reflect those of any organization that employs them.

## Footnotes

^1^Belgian Parliament. Belgian law on compulsory insurances for medical care coordinated on 14th July 1994 –art.72bis (Flemish: Wet Betreffende de Verplichte Verzekering Voor Geneeskundige Verzorging En Uitkeringen Gecoördineerd Op 14 Juli, 1994), Belgium.

^2^French Parliament. The Public health code. Article, R. 5124-49-1. Available online at: https://www.legifrance.gouv.fr/affichCodeArticle.do?cidTexte=LEGITEXT000006072665&idArticle=LEGIARTI000026428604&dateTexte=&categorieLien=cid

^3^Italian Medicines Agency. Available online at: http://www.agenziafarmaco.gov.it/it/content/carenze-dei-medicinali

^4^Spanish Agency for Medicines and Health Products. *Supply Shortages of Medicinal Products*. Available online at: https://www.aemps.gob.es/medicamentosUsoHumano/problemasSuministro/home.htm

^5^Regional Government of Madrid. *Medicamentos con problemas de suministro*. Available online at: http://www.madrid.org/cs/Satellite?cid=1142686373465&language=es&pagename=PortalSalud%2FPage%2FPTSA_pintarContenidoFinal&vest=1142645418945

^6^Government of Valencia. Law 1/2008, of April 17, on Guarantees for the Supply of Medicines. Article 3.

^7^Hungarian Parliament. *Act XCV/2005 (IDRAC 105308) on Human Medicines and the Amendment of Other Acts Regulating the Pharmaceutical Sector (“Medicines Act”)*.

^8^Federal Office of Public Health (FOPH). Available online at: https://www.bag.admin.ch/bag/en/home/themen/mensch-gesundheit/biomedizin-forschung/heilmittel/sicherheit-in-der-medikamentenversorgung.html

^9^Federal Office of National Economic Supply (FONES). Available online at: https://www.bwl.admin.ch/bwl/en/home.html

^10^Martinelli Consulting. Available online at: http://www.drugshortage.ch/index.php/uebersicht-2/

^11^Swiss Agency for Therapeutic Products − Swissmedic. Available online at: https://www.swissmedic.ch/swissmedic/en/home/humanarzneimittel/market-surveillance/out-of-stock.html

^12^Hungarian Professional College of Health Care. *Recommendations to Treat Drug Shortages (in Hungarian)*. Available online at: http://www.mgyt-kgysz.hu/index.php?option=com_docman&task=doc_download&gid=224

^13^The Irish Pharmacy Union. Available online at: https://ipu.ie/home/

^14^General Council of Provincial Pharmacy Chambers. *CISMED Database - Center for Information on Medicines Supply (in Spanish: Centro de Información sobre el Suministro de Medicamentos; CISMED)*. Available online at: http://www.portalfarma.com/Profesionales/consejoinforma/Paginas/Infarma-2016-CISMED.aspx

^15^Spanish Agency of Medicines and Health Products. *AEMPS Database*. Available online at: https://cima.aemps.es/cima/fichasTecnicas.do?metodo=buscarDesabastecidos

^16^Spanish Ministry of Health. *SEGUIMED*. Available online at: http://www.seguimed.es/seguimed-software-gestion-abastecimiento-medicamentos.html

^17^ELGLOBAL.NET. *El Seguimed es un sistema de control que está obsoleto*. Available online at: http://www.elglobal.net/hemeroteca/el-seguimed-es-un-sistema-de-control-que-esta-obsoleto-FYEG_888841

^18^Latvian Cabinet of Ministers. *Regulation No 416 of the Cabinet of Ministers of the Republic of Latvia “Procedures regarding the Distribution and Quality Control of Medicinal Products”* (adopted on 26 June 2007). Available online at: https://www.zva.gov.lv/?id=382&sa=382&top=333

^19^Turkish Minister of Health. Regulation: KHK/663; Item 27/Clause 2/Article d.

^20^European Parliament. Directive 2001/83/EC of the European Parliament and of the Council of 6 November 2001 on the Community code relating to medicinal products for human use.

^21^Albanian Council of Ministers. Bylaw no. 299 08.04.2015.

^22^State Agency of Medicines of the Republic of Latvia. Available online at: https://www.zva.gov.lv/?id=513&lang=&top=112&sa=377

^23^Polish Parliament. Ustawa z dnia 12 maja 2011 r. o refundacji leków, środków spozywczych specjalnego przeznaczenia zywieniowego oraz wyrobów medycznych (Act of 12 May 2011 on reimbursement of medicines, food products for special dietary use and medical devices).

^24^Polish Parliament. *Statute on Changing Statute on Pharmaceutical Law. Project of 21.03.2017 (Polish: ustawa o zmianie ustawy Prawo farmaceutyczne. Projekt 21.03.2017*). Available online at: https://legislacja.rcl.gov.pl/docs//2/12294213/12406989/12406990/dokument283459.pdf

^25^İTS (Pharmaceutical Track & Trace System). Turkey. Available online at: https://itsportal.saglik.gov.tr/index.php?run=content&get=27&mp=26,33

^26^King of Spain. Royal Decree 782/2013 on the Distribution of Medicines for Human Use (Article 3.3). Spain, 2013.

^27^Law 29/2006 of July 26. Article 101, c, 22 and Article 102. 1. Spain; 2006.

^28^COST Action CA 15105. COST Action CA 15105. *European Medicines Shortages Research Network - addressing supply problems to patients (Medicines Shortages*). Available online at: http://www.cost.eu/COST_Actions/ca/CA15105

^29^Canadian Generic Pharmaceutical Association. *Canadian Generic Pharmaceutical Association (CGPA) Best Practices Guidelines for the Prevention, Notification and Management of Drug Shortages*. Available online at: https://www.google.pl/url?sa=t&rct=j&q=&esrc=s&source=web&cd=1&cad=rja&uact=8&ved=0ahUKEwjw76ew9oHWAhXLPhQKHS4MD48QFggmMAA&url=http%3A%2F%2Fcanadiangenerics.ca%2Fwp-content%2Fuploads%2F2016%2F09%2FBestPracticesGuidelines2013.pdf&usg=AFQjCNHfkDUPTKfKbHV3Is

^30^GIRP European Association of Pharmaceutical Full-line Wholesalers. *European Association of Pharmaceutical Full-line Wholesalers (GIRP) response to the European Commission Consultation regarding Community action on Health Services Introduction*. Available online at: https://www.google.pl/url?sa=t&rct=j&q=&esrc=s&source=web&cd=7&ved=0ahUKEwjCxufIn4LWAhVBWhQKHTM6CAAQFghUMAY&url=http%3A%2F%2Fec.europa.eu%2Fhealth%2Farchive%2Fph_overview%2Fco_operation%2Fmobility%2Fdocs%2Fhealth_services_co76.pdf&usg=AFQjCNH2dxEuBukj_AoN

## Abbreviations

AEMPS: (in Spanish) Spanish Agency for Medicines and Health Products
AIFA: (in Italian) Italian Medicines Agency
ANSM: (in French) French Agency for Medicines Safety
CISMED: (in Spanish) Center for Information on Medicines Supply
EMA: European Medicines Agency
EOF: (in Greek) National Organization for Medicines
EU: European Union
FAMHP: Federal Agency of Medicines and Health Products (Belgium)
FONES: Federal Office of National Economic Supply (Switzerland)
GMP: Good Manufacturing Practice
MA: marketing authorization
MAH: Marketing authorization holder
MoH: Ministry/Minister of Health
NGO: Non-governmental organization
NIHDI: National Institute of Health and Disability Insurance (Belgium)
NoMA: Norwegian Medicines Agency
PSO: Public service obligation
SAMLV: State Medicines Agency in Latvia
SMCA: State Medicines Control Agency (Lithuania)
SOP: Standard operating procedures
TİTCK: Turkish Medicines and Medical Devices Agency.
