# Supplementary material for: Systemic Measures and Legislative and Organizational Frameworks Aimed at Preventing or Mitigating Drug Shortages in 28 European and Western Asian Countries
Source: Front Pharmacol. 2018 Jan 18;8:942. doi: 10.3389/fphar.2017.00942 (PMC5779072; doi:10.3389/fphar.2017.00942)
Supplement: Supplementary file 1 [file DataSheet1.docx]

***Supplementary Material***

**Systemic Measures and Legislative and Organizational Frameworks Aimed at Preventing or Mitigating Drug Shortages in 28 European and Western Asian Countries**

# Authors

Tomasz Bochenek*, Vafa Abilova, Ali Alkan, Bogdan Asanin, Iñigo de Miguel Beriain, Zeljka Besovic, Patricia Vella Bonanno, Anna Bucsics, Michal Davidescu, Elfi De Weerdt, Natasa Duborija-Kovacevic, Jurij Fürst, Mina Gaga, Elma Gailīte, Jolanta Gulbinovič, Emre Umut Gürpınar, Balázs Hankó, Vincent Hargaden, Tor Arne Hotvedt, Iris Hoxha, Isabelle Huys, Andras Inotai, Arianit Jakupi, Helena Jenzer, Roberta Joppi, Ott Laius, Marie-Camille Lenormand, Despina Makridaki, Admir Malaj, Kertu Margus, Vanda Marković-Peković, Nenad Miljković, João Luís de Miranda, Stanislav Primožič, Dragana Rajinac, David G. Schwartz, Robin Šebesta, Steven Simoens, Juraj Slaby, Ljiljana Sović-Brkičić, Tomas Tesar, Leonidas Tzimis, Ewa Warmińska, Brian Godman

***Correspondence**

Tomasz Bochenek MD, MPH, PhD, Department of Drug Management, Faculty of Health Sciences, Jagiellonian University Medical College, ul. Grzegorzecka 20, 31-531 Krakow, Poland, e-mail: mxbochen@cyf-kr.edu.pl

**SURVEY FORM**

Contributor’s name: …

E-mail address: …

Country: …

| **I.** | **General characteristics of the problem of drug shortages in your country** |
| --- | --- |
| I.1. | Is term “drug shortages” or its equivalent formally defined in your country? If YES - is there one or more different definitions of “drug shortages” co-existing in your country’s health care system? How precisely are “drug shortages” defined? Please, provide exact definition or definitions, together with references to the relevant legal or organizational documents where they are formulated? |
| Answer: | |
| I.2. | Has problem of drug shortages appeared in your country within the last decade?  (if your answer is NOT - please go directly to question no. I.8.) |
| Answer: | |
| I.3. | For how long has problem of drug shortages appeared in your country, when has it started? |
| Answer: | |
| I.4. | For which particular medicines is there a problem of drug shortages (international names and trade names, if possible)? |
| Answer: | |
| I.5. | With what frequency do shortages of particular medicines usually appear? |
| Answer: | |
| I.6. | What are average durations of particular medicine’s shortages? |
| Answer: | |
| I.7. | Has a problem of drug shortages increased or decreased within the past three years (if possible, please provide a more exact time frame)? |
| Answer: | |
| I.8. | Are there any statistics, “hard evidence” or other published or non-published records (registration system) on drug shortages in your country? If YES, please describe how reliable could they be and why. |
| Answer: | |
| I.9. | Space for any additional information or comments: |
| Answer: | |

| **II.** | **Alertness on drug shortages and description of information systems** |
| --- | --- |
| II.1. | Are pharmaceutical companies (Marketing Authorization Holders) formally obliged to notify a certain institution in your country in case of:   1. delayed or postponed commercialization of a medicinal product, 2. suspension, withdrawal or lack of renewal of marketing authorization, 3. predicted or sudden unavailability of a medicinal product due to other reasons, 4. cease of reimbursement of a medicinal product, 5. in other cases which could lead to drug shortages (when)?   If answer for either a) or b), c), d), e) is YES - please provide more details in point II.2. (below). |
| Answer: | |
| II.2. | What is the name of institution or organization which is being notified in cases stated above? What are their characteristics and channels for the above mentioned communication? |
| Answer: | |
| II.3. | Are there other institutions or organizations gathering signals and information on drug shortages (i.e. gathering them from sources other than the pharmaceutical industry) or running drug shortages databases? What are these institutions’ or organizations’ names and short characteristics? Are they the same or different than these mentioned in point I.8. of this questionnaire (above)? |
| Answer: | |
| II.4. | If any of answers I.8.; II.1.-II.3. (above) were positive, please provide details on rules of accessing, sharing and utilization of information on drug shortages. Who can access drug shortages information or database and how is this information shared with other stakeholders in health care system? |
| Answer: | |
| II.5. | Are there any “bottom-up” initiatives, working groups or committees, or informal networks which tackle the problem of drug shortages in your country? If YES, please describe them. |
| Answer: | |
| II.6. | Have there been any guidelines, codes of conduct, good practices or shortages management plans developed in your country aiming to manage the problem of drug shortages? If YES, please describe them. Who developed them? Have they been useful in practice? |
| Answer: | |
| II.7. | Space for any additional information or comments: |
| Answer: | |

| **III.** | **Public service obligations** |
| --- | --- |
| III.1. | Do the obligations exist in the health care system of your country, which are associated with supplying medicinal products to cover the needs of your country’s patients? If YES - which stakeholders have these obligations (pharmaceutical companies, MAH-s, wholesalers, others)? |
| Answer: | |
| III.2. | If the answer above was YES, do these obligations pertain to publicly reimbursed products only or to all pharmaceuticals from a given MAH’s or wholesaler’s portfolio? How are these obligations formulated? |
| Answer: | |
| III.3. | Have there been the above mentioned or any other public service obligations (PSO) described within your country’s legislation? If YES, which stakeholders of the pharmaceutical market have them and what is their kind or short characteristics? |
| Answer: | |
| III.4. | Space for any additional information or comments: |
| Answer: | |

| **IV.** | **Rules of medicinal products’ trade which could be associated with problem of drug shortages** |
| --- | --- |
| IV.1. | Can physicians order (prescribe) a medicinal product which is not registered (doesn’t have market authorization) in your country, so that it can be imported from country where it is registered? In which cases this is allowed? What’s the name for such procedure? |
| Answer: | |
| IV.2. | Can pharmacies or wholesalers order (import) a medicinal product which is registered in your country, from countries where it is also registered? If yes, in which cases? Does this apply only to situation when particular medicine is not available on the national market and there are no other treatment alternatives or maybe also to any other situations? |
| Answer: | |
| IV.3. | Which stakeholders (e.g. wholesalers, pharmacies – all of them or only the selected ones) can order medicines which are described in points IV.1. or IV.2. (above)? |
| Answer: | |
| IV.4. | Can parallel export of any medicine be limited or banned by any national institution? If yes, under which circumstances and by whom exactly? |
| Answer: | |
| IV.5. | Have there been any other measures developed in your country to impose pressure against excessive exportation of medicines? If yes, what are they specifically and who imposes them? |
| Answer: | |
| IV.6. | Space for any additional information or comments: |
| Answer: | |
